# Establishing a robust genetic sequencing and gene expression data library in cardiovascularly healthy cats

**DOI:** 10.1038/s41598-025-05704-8

**Published:** 2025-07-01

**Authors:** Joanna L. Kaplan, Victor N. Rivas, Jalena R. Wouters, Michael W. Vandewege, Samantha P. Harris, Joshua A. Stern

**Affiliations:** 1 Department of Medicine and Epidemiology, School of Veterinary Medicine, University of California-Davis, Davis, CA 95616 USA; 2 Feline Health Center, College of Veterinary Medicine, North Carolina State University, Raleigh, NC 27607 USA; 3 Department of Physiology, College of Medicine-Tucson, University of Arizona, 313 Medical Research Building, 1656 E Mabel St., Tucson, AZ 85724 USA

**Keywords:** Whole genome sequencing, RNA sequencing, Normal, Precision medicine, Individualized medicine, Hypertrophic cardiomyopathy, Genetics, Cardiology

## Abstract

Cardiovascular disease is a leading cause of increased morbidity and mortality in cats, with hypertrophic cardiomyopathy (HCM) significantly overrepresented. While feline HCM is hereditary, the genetic etiology of disease remains poorly understood. Establishing a cohort of well-phenotyped and -genotyped healthy control cats is essential to fuel future genetic/pharmacogenetic discoveries. We sought to construct a robust genetic sequencing and gene expression library from cardiovascularly healthy cats using whole genome sequencing (WGS) and RNA sequencing (RNA-Seq). Fifty-four client-owned cats ($\ge$ 10 years) were screened, of which 18 cats (cohort 1) were prospectively enrolled after being deemed cardiovascularly healthy by clinicopathology, biochemistry, and echocardiography. DNA isolated from blood samples was submitted for paired-end WGS at ~ 30X coverage. Standard pipelines were employed for variant calling across sequenced cats. A second cohort of 15 purpose-bred cats were euthanized for non-cardiac reasons. Flash-frozen left ventricular (LV), interventricular septum (IVS), and left atrium (LA) tissues from 11, 14, and 13 cats, respectively, were submitted for stranded mature RNA-Seq at 50 million reads/sample. Gene variants and expression profiling were catalogued for both meticulously selected cohorts. Transcriptomic and WGS data libraries were generated to serve as an open-access resource in future investigations of feline cardiovascular precision medicine.

**Supplementary Information:**

The online version contains supplementary material available at 10.1038/s41598-025-05704-8.

## Introduction

Cardiovascular disease is a significant cause of increased morbidity and mortality in the feline population^1–5^. A 2009 study investigating causes of mortality in a large Swedish cohort of life-insured and mainly pure-bred cats ranked cardiovascular disease among the top five leading causes of death of which cardiomyopathy was the most commonly reported^1^. In addition, a 2015 study evaluated the longevity of pure-bred and mixed-breed cats visiting primary care facilities in England and listed cardiac disease as the eighth most frequently reported cause of mortality^2^. Among cardiovascular diseases, hypertrophic cardiomyopathy (HCM) is undoubtedly the most pervasive in the domestic cat with a reported prevalence as high as 15%^6^, while congenital cardiac disease and other cardiomyopathies have a reported prevalence of 0.14–0.5%^6–8^, and 0.1%^6^, respectively.

In humans, large advancements in genetic discovery have aided in investigating the complex pathophysiology of HCM, allowing for improved screening practices, risk stratification protocols, and the development of targeted novel drug therapies^9–11^. The current veterinary literature extensively characterizes feline cardiac disease, paying particular attention to HCM using both antemortem and post-mortem techniques^5,12,13^. Similar to observations in people, feline HCM is considered largely hereditary^12,14–17^. Thus far, six genetic mutations have been described to be associated with development of HCM in cats, but only two have withstood replication and are currently classified as pathogenic when the American College of Medical Genetics and Genomics guidelines are applied^18^. The two pathogenic classified variants include the A31P mutation in Maine Coon cats and the R820W mutation in Ragdolls, both of which lie within the *MYBPC3* gene encoding the sarcomeric protein, cardiac myosin binding protein C^18^. However, all previously identified mutations are breed-specific and do not explain the underlying cause of HCM in the general cat population^5,12^. While limited genetic discovery has already led to improved breeding and screening practices within these specific cat breeds, the vast heterogeneity in disease phenotype, severity, and outcome has made it difficult to understand the exact underlying molecular and genetic pathways involved in disease pathogenesis^19–22^. In addition, the genetics associated with more rare cardiac diseases in the general cat population remain poorly understood. This large and seemingly impenetrable knowledge gap has likely stunted our ability to identify disease early, effectively prognosticate, and foster new drug discovery and treatment strategies.

Continued advancements in whole genome sequencing (WGS) allow for more in-depth and comprehensive detection of variants that either cause disease or influence disease severity and outcome^22–24^. Furthermore, the addition of RNA sequencing (RNA-Seq) allows for the ability to explore gene expression within tissues and molecular pathways involved in disease pathogenesis. An essential component to new genetic discovery is generating an appropriate cohort of phenotypically normal cats to compare to disease-affected subjects. Identifying these normal cats can be of great challenge when ruling out diseases like HCM that have a significant component of age-related penetrance. The objective of this study is to curate a robust genetic sequencing and gene expression data library from cardiovascularly healthy cats to serve as a valuable, free, and open-access tool for use in future genetic investigations of feline individualized medicine.

## Results

### Cohort 1

**Fig. 1:**
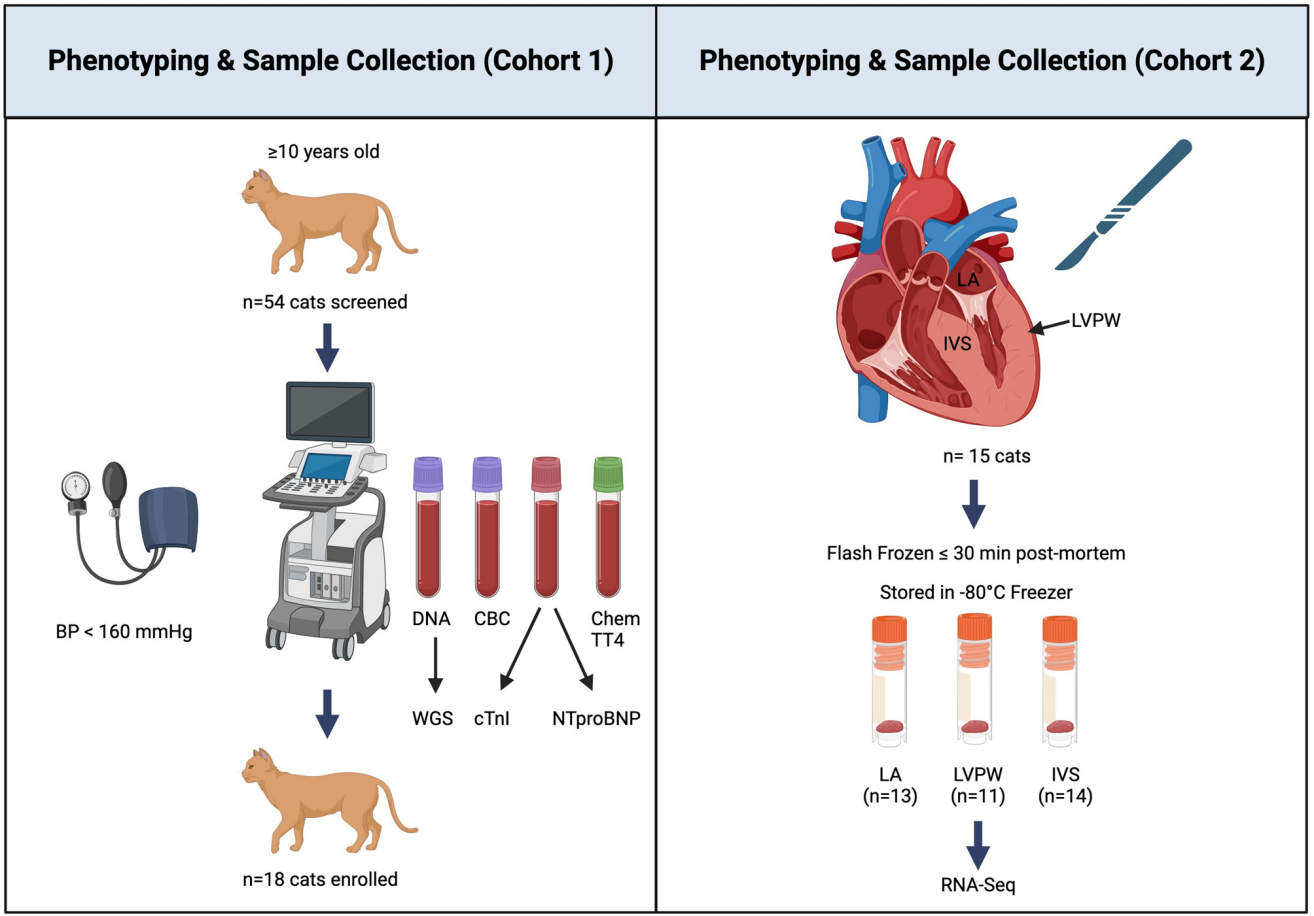
Graphical depiction of study design for cohorts 1 (left) and 2 (right).

Whole blood samples from cardiovascularly-healthy, client-owned, geriatric cats ($\ge$ 10 years) were collected for DNA isolation and submission for WGS (Fig. 1). Cats were deemed cardiovascularly healthy on clinicopathology, biochemistry, and echocardiography. A total of 54 cats were recruited for cohort 1. Thirteen cats were excluded for an echocardiographic diagnosis of HCM phenotype, of which one cat had concurrent systemic hypertension (BP > 200 mmHg) and one cat had a concurrent elevation in total thyroxine (T4). Four cats were excluded for presence of equivocal-to-mild LA dilation of which one had a concurrent elevation in T4, one had a concurrent right bundle branch block (RBBB), and one had ventricular trigeminy. Three cats were excluded for equivocal LV wall thickening, of which one had intermittent axis deviation on a single-lead II electrocardiogram (ECG) that was not further characterized with a 6-lead ECG. Five cats were excluded for an elevated creatinine, of which one had concurrent axis deviation on ECG, one had ventricular ectopy and left anterior fascicular block, and one had concurrent pleural effusion identified on echocardiogram. Two additional cats were excluded for isolated elevations in T4. One cat was excluded for a mass-like lesion along the atrioventricular groove adjacent to the tricuspid valve, one cat was excluded for the presence of a cardiomyopathy of non-specific phenotype with concurrent ventricular premature complexes, and five cats were excluded for the presence of a left anterior fascicular block confirmed on a 6-lead ECG. Two cats were excluded after completion of the study after follow-up echocardiography revealed mild HCM. Eighteen cats remained and were successfully enrolled in the study for WGS.

Of the remaining 18 cats, breeds included domestic short hair (12), domestic long hair (2), and one of each of the following: Persian, Maine Coon, Siberian, and a Siamese cross. Nine cats were female spayed and nine cats were male castrated. Six out of 18 cats had a cardiac murmur. Of these cats, 4/6 had a grade II/VI right apical systolic murmur, three of which were confirmed via echocardiography to be consistent with a dynamic right ventricular outflow tract obstruction (DRVOTO). Two out of six cats had a grade II/VI left parasternal systolic murmur presumed to be physiologic due to the lack of any structural change identified on echocardiogram. In the remaining cats with heart murmurs, a dynamic left or right ventricular outflow tract obstruction could not be documented during the echocardiogram. The remaining 12 cats had no auscultatory abnormalities. On a single-lead II ECG during the echocardiogram, two cats had evidence of a RBBB, which was subsequently confirmed on a 6-lead ECG. Spectral Doppler of transmitral flow revealed fused E and A waves in 10/18 cats (one of which had a normal pattern of relaxation on pulse wave tissue Doppler imaging). In cats with separation of E and A waves on Spectral Doppler of transmitral flow, a normal relaxation pattern was observed in 3/18 cats, and an impaired relaxation pattern was observed in 5/18 cats (two of which showed a pseudonormal relaxation pattern). Additional clinical, echocardiographic, and labwork variables of cats undergoing WGS are listed in Table 1 and supplementary Tables S1 and S2.

**Table 1:** Clinical, laboratory, and echocardiographic variables in WGS cohort of cats.

Variables	Number of cats (n)	Mean (SD) or Median (IQR)
Clinical Variables		
Age (years)	18	12 (11,13)
Body Weight (kg)	18	5.4 (1.0)
Heart Rate (bpm)	18	184.3 (21.6)
Blood Pressure (mmHg)	18	136.7 (11)
Laboratory Variables		
Creatinine (mg/dL)	18	1.36 (0.24)
BUN (mg/dL)	18	24.61 (5.17)
Total T4 (ug/dL)	18	3.18 (0.45)
NTproBNP (pmol/L)	18	58.17 (34.47)
cTnI (ng/L)	18	34.78 (19.47)
Echo Variables		
LAD_2D RPLx4c_ (mm)	18	13.2 (1.5)
LA_RPSx_ (mm)	18	12.41 (1.13)
Ao_RPSx_ (mm)	18	9.40 (8.80,10.10)
LA:Ao_RPSx_ (mm)	18	1.3 (0.1)
LVIDd_RPSx MM_ (mm)	18	14.88 (1.47)
LVIDs_RPSx MM_ (mm)	18	6.54 (1.82)
FS_RPSx MM_ (%)	18	56.40 (10.28)
IVSd Max (mm)	18	5.41 (0.55)
LVPWd Max (mm)	18	5.28 (0.57)
Max LV Thickness (mm)	18	5.81 (5.16,5.85)
LVOT_Vmax_ (m/s)	16	0.90 (0.21)
LAA Flow Velocity (cm/s)	18	57.51 (19.01)

BUN, blood urea nitrogen; T4, thyroxine; NTproBNP, N-terminal B-type natriuretic peptide; cTnI, cardiac troponin I; LAD, left atrial diameter; LA, left atrium; Ao, aortic root; LVIDd, left ventricular internal dimension at end-diastole; LVIDs, left ventricular internal dimension at end-systole; FS, fractional shortening; IVSd, interventricular septum at end-diastole; LVPWd, left ventricular free wall at end-diastole; LVOT, left ventricular outflow tract, LAA, left atrial appendage; RPLx4c, right parasternal long axis four chamber; RPSx, right parasternal short axis; MM, M-mode; V_max_, maximal velocity. Testing for normality was performed using a D’Agostino & Pearson Test. Normally and non-normally distributed data was reported as the mean and standard deviation (SD) or median and interquartile range (IQR), respectively.

**Fig. 2:**
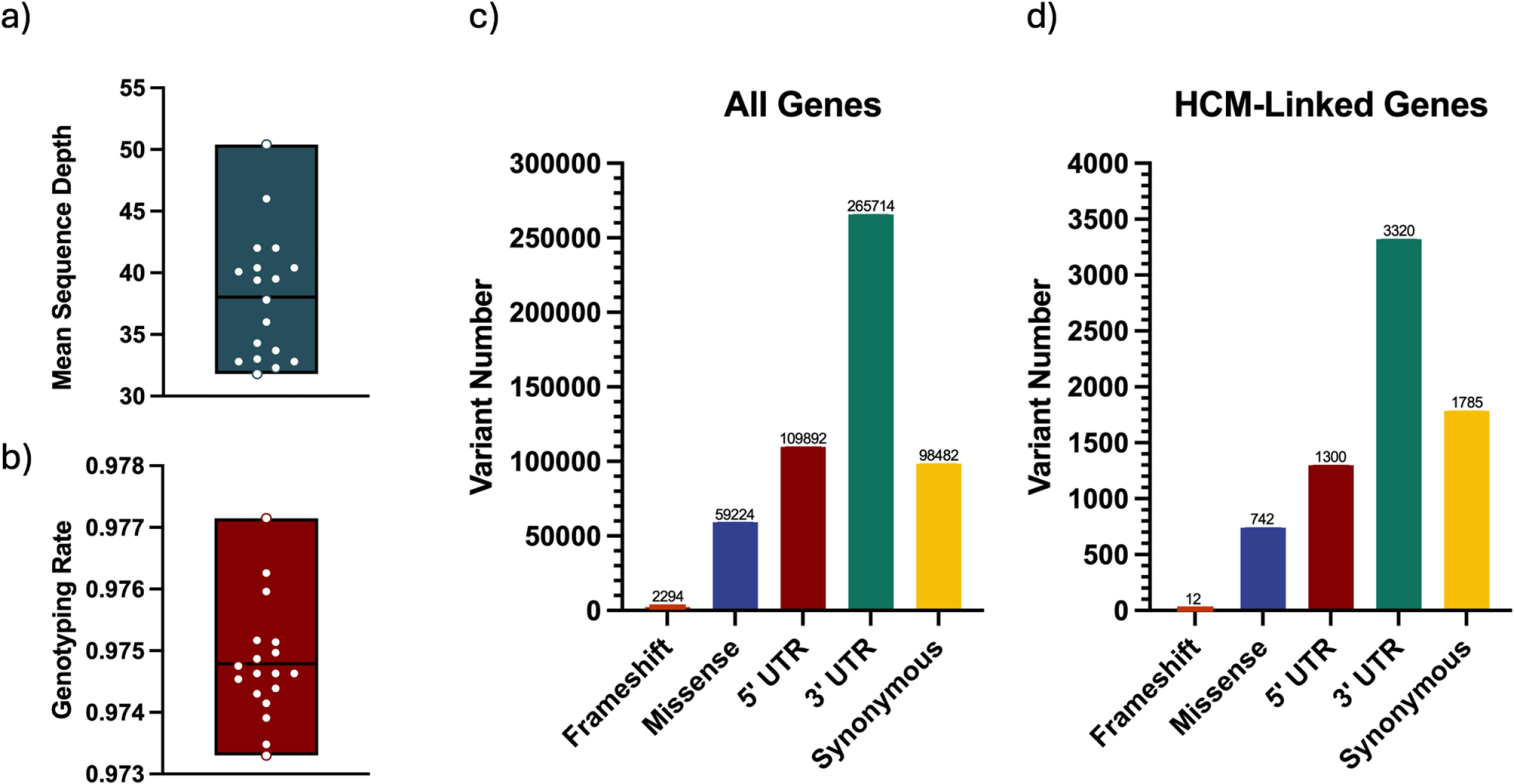
(**A**) Average sequencing depth among samples in cohort 1, (**B**) proportion of variants that were genotyped in each individual, (**C**) total number of common genic variants identified categorized predicted among all genes, and (**D**) total number of common genic variants identified categorized predicted among those found in HCM-linked genes.

Whole genome sequencing was successfully performed in all 18 cats and deposited to NCBI’s SRA under Bioproject PRJNA1160267 (supplementary Table S1). Paired-end reads were aligned to the F.catus_Fca126_mat1.0 reference genome. The average sequencing depth was 38x (Fig. 2A). Overall, 35,248,385 variants were discovered with a transition/transversion rate of 2.25, within the mammalian range, and the genotyping rate was above 97% (Fig. 2B) for all cats. These metrics indicate high-quality sequence data. Figure 2C is a graphical depiction of the most common genic variants overall, as well as variants identified within genes associated with the development of HCM in human beings (Fig. 2D).

A total of 265,714 3′untranslated region (UTR) variants, 109,892 5′UTR variants, 98,482 synonymous variants, 59,224 missense variants, and 2294 frameshift variants were identified overall. We performed a search of the ClinVar human database for variants and genes linked to hypertrophic cardiomyopathy (Accessed 31 May 2024). A total of 24,313 variants within 246 unique genes were identified in this search. Within our cohort of cats, a total of 3320 3′UTR variants, 1300 5′UTR variants, 1785 synonymous variants, 742 missense variants, and 12 frameshift variants were identified within 207 of these genes associated with HCM in people.

### Cohort 2

**Fig. 3:**
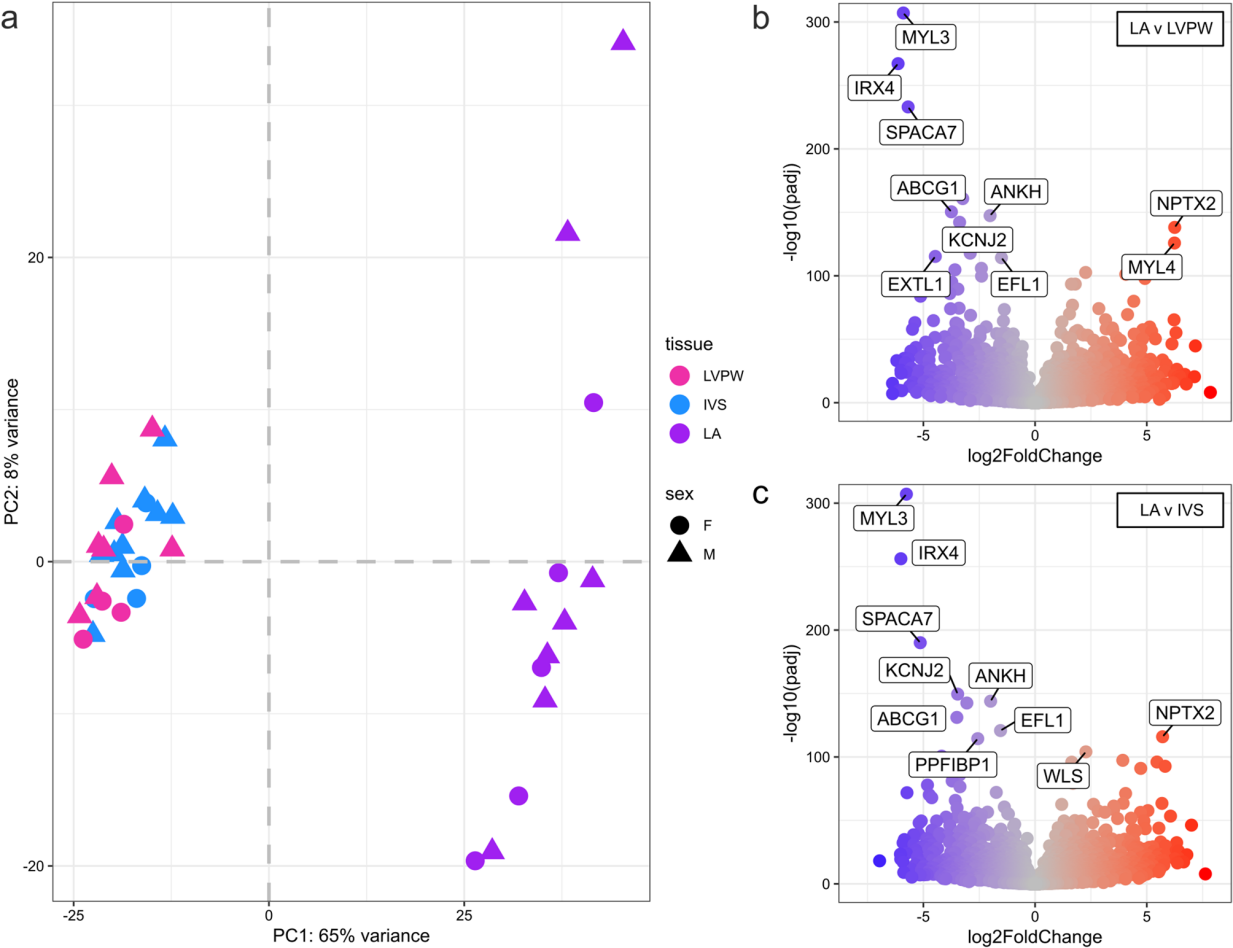
(**A**) Principal component analysis (PCA) plot demonstrating gene expression variance among the left ventricular posterior wall (LVPW), interventricular septum (IVS), and left atrial (LA) tissue. Triangles and circles represent male and female cats. (**B**) Volcano plot indicating up- and down-regulated DEGs in LVPW tissue when compared to LA tissue. (**C**) Volcano plot indicating the up- and down-regulated DEGs in IVS tissue when compared to LA tissue. DEGs with the lowest 10 p-values are labeled in (**B**) and (**C**).

Cohort 2 consisted of a convenience sample of 15 purpose-bred cats euthanized for non-cardiac reasons. All cats were apparently healthy without any therapeutic interventions. Immediately flash-frozen left ventricular posterior wall (LVPW), interventricular septum (IVS), and left atrium (LA) tissues from 11, 14, and 13 cats, respectively, were collected and submitted for RNA-Seq. Clinical and echocardiographic variables, as well as NCBI accession numbers for transcript data for cats in cohort 2 are listed in Table 2 and supplementary Tables S3 and S4. Table 3 lists the number of differentially expressed genes (DEGs) that were upregulated and downregulated between tissue types. There were no differences in DEGs between the IVS and LVPW. However, when LA tissue was compared to LVPW tissue, a total of 1974 genes were upregulated and 834 genes were downregulated in LA tissues. A total of 2161 genes were upregulated and 935 tissues were downregulated in the LA tissue when compared to IVS tissue. Little differences were noted in tissues between female and male cats and when cats were categorized as above or below 5 years of age. Up to the top 25 upregulated and downregulated DEGs for each tissue comparison are listed in supplementary Tables S5–S14. In addition, the top 25 genes expressed in each tissue overall are listed in supplementary Tables S15–S17. Supplementary Table S18 shows a compilation of genes listed in supplementary Tables S15–S17 to demonstrate overlap between LA, LVPW, and IVS tissues. Figure 3A demonstrates the expression variance among tissue types with notable differences between LA tissues compared to LVPW and IVS tissues, but no substantial differences between LVPW and IVS tissues. Consistently, there were no DEGs between IVS and LVPW (Table 3). Figure 3B and 3C demonstrate the top upregulated and downregulated DEGs in LA tissue when compared to LVPW and IVS tissue, respectively. Overall, DEGs were relatively similar in IVS and LVPW compared to LA (supplementary Tables S11–S4). Of note, the sarcomeric gene *MYL3,* cell adhesion gene *SPACA7*, and transcription factor *IRX4* are upregulated in both LVPW and IVS tissue in comparison to LA tissue. In addition, the sarcomeric gene *MYH7* is upregulated in LVPW and IVS tissue compared to LA tissue. In contrast, *MYL4*, which encodes for an atrial-specific myosin light chain, is upregulated in the LA as expected compared to the LVPW and IVS (Fig. 3B; supplemental Tables S12; S14).

**Table 2:** Clinical and echocardiographic variables in RNA-Seq cohort of cats.

Variables	Number of cats (n)	Mean (SD) or median (IQR)
Age at time of death (years)	15	5.8 (3.3)
Weight (kg)	10	4.18 (0.79)
LAD_2D RPLx4c_ (mm)	14	12.61 (1.22)
LA_RPSx_ (mm)	12	11.33 (1.57)
Ao_RPSx_ (mm)	12	8.58 (1.02)
LA:Ao_RPSx_ (mm)	12	1.32 (0.15)
LVIDd_RPSx MM_ (mm)	14	14.03 (2.48)
LVIDs_RPSx MM_ (mm)	14	7.83 (2.12)
FS_RPSx MM_ (%)	14	44.89 (8.26)
IVSd Max (mm)	15	5.2 (0.50)
LVPWd Max (mm)	15	5.16 (0.38)
Max LV Thickness (mm)	15	5.45 (0.31)
LVOT_Vmax_ (m/s)	13	0.73 (0.13)
LAA Flow Velocity (cm/s)	11	42.42 (12.09)

LAD, left atrial diameter; LA, left atrium; Ao, aortic root; LVIDd, left ventricular internal dimension at end-diastole; LVIDs, left ventricular internal dimension at end-systole; FS, fractional shortening; IVSd, interventricular septum at end-diastole; LVPWd, left ventricular free wall at end-diastole; LVOT, left ventricular outflow tract, LAA, left atrial appendage; RPLx4c, right parasternal long axis four chamber; RPSx, right parasternal short axis; MM, M-mode; V_max_, maximal velocity. Testing for normality was performed using a D’Agostino & Pearson Test. Normally and non-normally distributed data.

**Table 3:** Number of upregulated and downregulated differentially expressed genes (DEGs) between the following tissue comparisons were made: interventricular septum (IVS) versus left ventricular posterior wall (LVPW), left atrial (LA) versus LVPW, LA versus IVS, IVS from male versus female cats, LA from male versus female cats, LVPW from male versus female cats, IVS in cats $\ge$ 5 yrs of age versus < 5 years of age, LA in cats $\ge$ 5yrs of age versus < 5 years of age, LVPW in cats $\ge$ 5 yrs of age versus < 5 years of age.

Tissue Comparisons	Upregulated DEGs	Downregulated DEGs	Total DEGs
IVS versus LVPW	0	0	0
LA versus LVPW	2161	935	3096
LA versus IVS	1974	834	2908
IVS_Male_ versus IVS_Female_	2	5	7
LA_Male_ versus LA_Female_	2	2	4
LVPW_Male_ versus LVPW_Female_	35	9	44
IVS_≥5yrs_ versus IVS_<5yrs_	1	1	2
LA_≥5yrs_ versus LA_<5yrs_	2	0	2
LVPW_≥5yrs_ versus LVPW_<5yrs_	16	2	18

Numbers of DEGs represent the log2 of the first tissue listed over the second.

Gene ontology (GO) enrichment analysis for cellular components (CC), biological processes (BP), and Kyoto Encyclopedia of Genes and Genomes (KEGG) pathways for upregulated and downregulated DEGs within the IVS and LVPW tissues compared to the LA tissues, as well upregulated DEGs within the LVPW tissue in male cats compared to female cats are shown in supplementary Figs. S1–S3, respectively. Up to the top 20 terms identified for each are reported. The majority of GO BP, CC, and KEGG terms identified within IVS and LVPW tissue when compared to LA tissue are consistent with energy and fatty acid metabolism, cardiac muscle contraction, and genes previously associated with cardiomyopathies. Of note, pathways in complement and coagulation cascades and platelet activation are upregulated in LA tissue compared to IVS and LVPW tissue, respectively.

## Discussion

We have created an open-access collection of transcriptomic and WGS data from 15 purpose-bred and 18 client-owned cardiovascularly healthy cats, respectively. These collections can be accessed via the links provided in supplementary Tables S1 and S3. The two cohorts of cardiovascularly normal cats provided in this study have undergone extensive phenotyping to serve as healthy controls for future studies in genetic discovery and precision medicine that aim to improve early identification and management of feline cardiovascular disease.

Out of 54 cats originally screened, only 18 cats were successfully enrolled in cohort 1. This exposes the great difficulty in identifying older cats that are truly free of cardiovascular disease and other systemic comorbidities. However, establishing a well-phenotyped control group is imperative to identifying disease-causing variants. Currently, the 99 Lives consortium provides an open-access collection of over 300 domestic cat genomes and has proved to be an invaluable resource in genetic discovery investigations^23^ (Buckley and Lyons VCSA 2020). However, the cardiovascular disease status or phenotype is not reported in the vast majority of cats within this collection. Therefore, our cohort fills a significant gap by providing detailed clinical, hematological, and echocardiographic findings in the largest open-access WGS data collection of cardiovascularly normal cats. While the sample size remains small and may limit its application for certain applications, the authors believe the value of this validation cohort remains critical for future feline cardiovascular genetic discovery.

Of interest, our WGS data identified a large number of variants in our healthy cohort of cats previously demonstrated to be associated with HCM-linked genes in humans^25^. This finding is crucial as studies that lack an adequate control group may incorrectly assume that variants identified in genes linked to human HCM are also pathogenic in the cat when instead, the variant of interest could be of high prevalence in a healthy cat population and is of no clinical consequence. The cohort presented in this study will likely minimize the risk of future misclassification of clinically silent variants of unknown significance as pathogenic. An alternative explanation is that our findings may instead highlight the heterogeneous nature of HCM. For example, there may truly be disease-causing or disease-modifying variants in our control population that have simply not been phenotypically expressed in this cohort due to semidominance or incomplete penetrance^14,26,27^.

As expected, we found statistically significant differences in upregulated and downregulated DEGs in IVS and LVPW tissues compared to LA tissues. These findings are consistent with reports in humans and a previous study evaluating DEGs between 5 LV samples and 5 LA samples from 1.5 year old healthy male cats^28–30^. Consistent with the latter report, *MYL3* and *IRX4* were all found to be significantly upregulated in healthy feline LVPW and IVS tissue compared to LA tissue. In addition, *MYH7* was found to be significantly upregulated in LVPW tissue. *MYL3* encode essential myosin light chain while *MYH7* encodes beta-myosin heavy chain, all of which have had genetic mutations identified and associated with human HCM^9^. *IRX4* (Iroquois-related homeobox gene) has previously been identified to be crucial in the embryological development of the ventricle by activating expression of ventricle myosin heavy chain-1 (*VMHC1*) and downregulating expression of atrial myosin heavy chain-1 (*AMHC1*)^31^. While the previous study in healthy cats evaluated LV tissue as a whole rather than exploring DEGs in LVPW and IVS separately, it is interesting to note that we found no differences in DEGs between these tissues. This finding is consistent with reports in healthy human heart tissue showing small to no regional differences between LVPW and IVS tissues^28,32^. However, it is unknown if this finding would remain consistent in diseased LV tissue, particularly in diseases that lead to asymmetrical structural changes such as feline HCM.

We also identified the top 25 most abundantly expressed genes overall from each tissue type and compiled them into supplementary Table S18. This helped to identify highly expressed genes within all tissues that were not necessarily chamber-specific. Where there was no overlap, *EEF1A1*, *MYH6*, *MYL4*, *MYL7*, *NPPA*, and *PAM* were noted to be abundantly expressed in LA tissue but not in LV or IVS tissues. In contrast *ACTA1*, *MYBPC3*, *MYH7*, *MYL2*, *MYL3*, and *PLN* were abundantly expressed in both IVS and LVPW tissues but not in LA tissue. The *NPPA* gene codes for the precursor of atrial natriuretic peptide (ANP) and was previously documented to have higher expression in the atria compared to the ventricles in humans^33–35^. In addition, a study in mice showed that *PAM*, which codes for peptidylglycine alpha-amidating monooxygenase, plays an essential role in formation of atrial secretory granules^36^. Consistent with that observed in our cohort of cats, the genes that code for alpha myosin heavy chain (*MYH6*) and atrial-specific myosin light chains (*MYL4* and *MYL7*) are more abundantly expressed in human LA tissue, whereas the genes that code for beta myosin heavy chain (*MYH7*) and ventricular-specific myosin light chains (*MYL2* and *MYL3*) have increased expression in LV tissue^35,37^. This is in contrast to mice, a common small animal translational model, in which alpha myosin heavy chain is the predominant isoform in ventricular tissues^38,39^. Interestingly, *EEF1A1*, a key player in protein synthesis, was more abundantly expressed in LA tissue in our cohort of cats compared to LV tissue^40^. This contrasts with findings in human tissue, in which *EEF1A1* is expressed most abundantly in non-cardiac tissues (i.e., brain, placenta, lung, liver, kidney, and pancreas) and is almost nondetectable in heart tissue^41^. In addition, *ACTA1*, which codes for skeletal actin, is co-expressed with *ACTC1* in the human adult heart and has not been shown to be cardiac chamber specific. These differences in gene expression may be reflective of species differences. Alternatively, it is possible that these genes are highly expressed in the feline left ventricle, as well, but did not make the top 25 list. Our overall findings demonstrate that when compared to mice healthy feline heart tissues exhibit similar gene expression compared to their human counterparts, reinforcing the value of the domestic cat as a large animal translational model of cardiac disease in human beings. Understanding which genes are either non-specific or specific to the atria or ventricles in the cat and how this compares to that of human heart tissue may be of particular importance when trying to identify novel cardiac tissue-specific drug targets that may benefit both the feline population and their human counterparts. This data may also be of value for narrowing the search for genetic variants associated with feline cardiac disease in future studies.

Our cohorts of control cats have already proven invaluable in its application in precision medicine both in clinical practice and future genetic discovery work. A report of a 21-month-old cat that presented to the UC Davis Veterinary Medical Teaching Hospital for recurrent episodes of respiratory distress utilized WGS data reported herein to identify a novel c.1986_1987insCC autosomal recessive variant in *ITGA2B*^42^*.* Identification of this mutation led to the subsequent diagnosis of Glanzmann’s thrombasthenia so that the most appropriate management and treatment strategy could be implemented for that patient in real time. We anticipate similar success with cardiovascular genotyping using strong cohorts of control cats.

In addition, our control cohorts are currently being used in a large-scale multiomics study involving WGS and RNA-Seq of over 100 HCM-affected cats of all breeds to further characterize the genetics of HCM and better understand the interrelationship between genotype, clinical phenotype, and outcome. While HCM is the most common cardiovascular disease observed in cats, we predict that this cohort will also serve useful to investigate the genetics of rarer acquired and congenital feline cardiac diseases that have been largely underexplored in the literature^43^.

Precision medicine is a growing focus in both human and veterinary medicine^22,24,44^. Rather than utilizing a one-size-fits-all approach, it involves designing a tailored diagnostic and management plan based on a deep understanding of the genetic and molecular pathways that are unique to the disease pathogenesis of each individual patient. Personalized medicine often requires a multiomics approach, integrating medical history, physical exam findings, and diagnostics with genomics, proteomics, and metabolomics data. Precision medicine has an exciting potential to significantly advance the management of feline cardiovascular disease^22–24^. However, a greater understanding of the intricate genetic and cellular interactions involved in phenotypic expression of disease is essential to improve our ability to prognosticate disease outcome and create targeted novel drug therapies^22,45^. The highly heterogenous nature of many feline cardiovascular diseases makes the advancement of precision medicine particularly challenging while simultaneously underscoring its importance.

Although arguably in its infancy in veterinary practice, precision medicine has already significantly impacted management of human and feline cardiovascular disease^10,21,24^. Specifically, the discovery of molecular pathways involved in HCM-associated pathologic hypertrophy has led to the development of several novel therapies^10,11,21,38,46–51^. One important example of this is the development of small-molecule inhibitors, mavacamten and aficamten. The myosin binding protein C encoding gene, *MYBPC3*, is one of the most commonly identified genetic causes of HCM in people^9,10^, and has been identified to cause HCM in the Maine Coon and Ragdoll breeds^15,16^. In vivo studies in mice demonstrated that causative mutations in the *MYBPC3* gene may alter the super relaxed state of myosin, a possible explanation for poor myocardial energetics, hypercontractility and resultant compensatory hypertrophy, diastolic dysfunction, fibrosis, and arrhythmias^46–48^. Utilizing this knowledge, a firstin-class small-molecule inhibitor, mavacamten, was designed to directly target the myosin ATPase to reduce contractility and relieve left ventricular outflow tract obstruction (LVOTO)^10,11,38,51^. Shortly thereafter, a second small-molecule inhibitor, aficamten, was developed for the same purpose. Both compounds were evaluated in purpose-bred cats with the A31P *MYBPC3* mutation along their pathway to launch human clinical trials and seek FDA approval for humans with obstructive cardiomyopathy^11,21,49–52^. These findings suggest that future genetic discoveries in feline HCM and pharmacogenomic work could help elucidate our understanding of drug-disease interactions.

In addition to small-molecule inhibitors, a new formulation of delayed release (DR) rapamycin is currently being explored for treatment of subclinical feline HCM^53^. Rather than targeting the sarcomere, this drug targets the mTOR pathway, a key pathway involved in pathologic cardiac remodeling. Results of a recent prospective study demonstrated that this new formulation may delay or prevent future pathologic LV remodeling in cats with subclinical HCM. Our control cohorts could be instrumental in understanding what genes, if any, cause dysregulation of the mTOR pathway in feline HCM to help identify specific populations of HCM-affected cats that would derive maximal benefit from this drug.

Despite our best efforts, there are inherent limitations to this study. In cohort 1, we specifically only enrolled cats $\ge$ 10 years of age to make the possibility of developing HCM or acquired cardiomyopathy exceedingly unlikely. Despite this age cut-off, two of our cats initially enrolled were excluded 10 and 11 months after study completion due to development of HCM on follow-up echocardiograms. This limitation likely emphasizes the difficulty in establishing a control group for complex heterogenous diseases with variable ages of onset. In addition, two cats in cohort 1 had NTproBNP values above the reference limit. After careful consideration, we decided to not exclude them given their normal echocardiogram and continuous ECG findings. One previous study identified that quantitative evaluation of NTproBNP was an effective screening tool for occult feline cardiomyopathy with a sensitivity of 71% and specificity of 100% at a cutoff of > 99 pmol/L. However, that study acknowledged that the rate of false positives may have been lower than expected in the general cat population due to the high prevalence of disease in their tertiary referral study population^54^. A second study evaluating the utility of using NTproBNP in screening for occult HCM demonstrated similar results with an NTproBNP cut-off value of > 100 pmol/L and a sensitivity and specificity of 92.4% and 93.9%, respectively^55^. Therefore, although uncommon, that study identified cardiovascularly normal cats with an NTproBNP within a range of 100–150 pmol/L. With the results of these two studies in mind, we decided to include the two cats with elevated NTproBNP values in our healthy cohort 1. However, it should be noted that all laboratory findings for each cat are provided in supplementary Table S1 so that future researchers may decide at their own discretion which cats to include in future investigations based on the particular aims of any one study. Long-term follow-up of these two cats will be required to determine if they develop cardiac disease after study completion. While the use of cats greater than 10 years of age is critical to minimize the risk of future cardiovascular disease development, it may also introduce a longevity bias that should be considered for future use of this dataset.

Another important note to highlight is that four cats in cohort 2 were heterozygous for the *MYBPC3* mutation. However, the results of RNA-Seq depict their gene expression only at the time of euthanasia. Therefore, despite their genotype, these four cats were not expressing a clinically recognizable phenotype of HCM at the time of euthanasia. Multiple clinical studies have documented that client-owned cats heterozygous for the A31P mutation have no increased risk of HCM development^20^. However, it is possible that gene expression may be altered before our ability to detect HCM echocardiographically or histologically. In addition, purpose-bred cats from the A31P colony that were wild-type (WT) for the A31P *MYBPC3* mutation may harbor unidentified disease modifier genes that could alter gene expression and therefore, not be entirely reflective of gene expression in a more heterogenous domestic cat population. Finally, the RNA-Seq data provided for cohort 2 also covers a comparatively broader ranges of age compared to the solely geriatric data of cats in cohort 1. While this likely makes the resultant data of relevance to a broader range of future research, it may also represent a limitation if attempting to apply the data obtained from both cohorts 1 and 2 simultaneously. Because each data set and all demographic and relevant data are provided for each case of each cohort, we hope that future investigators will be able to use this dataset in an *a la carte* method when the study design dictates it.

In conclusion, we have created an open-access collection of transcriptomic and WGS data from cardiovascularly normal cats that have been extensively phenotyped using several diagnostic modalities, including Doppler blood pressure (BP), echocardiography, ECG, systemic bloodwork, and blood biomarkers of cardiovascular disease. To the authors’ knowledge, this is the largest collection of WGS and RNA-Seq data from cats confirmed to be free of cardiovascular disease. This will likely serve as an invaluable tool in future genetic discovery work and investigations of personalized feline cardiovascular medicine.

## Methods

Two separate cohorts of healthy control cats were recruited for this study. Study procedures were approved by the Institutional Animal Care and Use Committee at the University of California, Davis (protocols #21857 and #22376) and in compliance with the ARRIVE2.0 guidelines^56^, the Animal Welfare Act, and the Institute for Laboratory Animal Research Guide for the Care and Use of Laboratory Animals.

### Cohort 1

#### Animals

Apparently healthy cats $\ge$ 10 years of age were prospectively recruited from clients and the veterinary community at the University of California, Davis, William R. Pritchard Veterinary Medical Teaching Hospital (Fig. 1). Written client-informed consent was obtained for all subjects prior to enrollment. All cats underwent a routine physical examination, Doppler sphygmomanometry-assessed systolic BPBP, complete echocardiogram, and phlebotomy for a complete blood count (CBC), biochemistry panel, total serum thyroxine (T4) level, serum high-sensitivity cardiac troponin I (cTnI), and serum N-terminal pro-B-type natriuretic peptide (NTproBNP). Cats were excluded if they had any evidence of structural congenital or acquired cardiovascular disease on echocardiogram. All cats had a continuous lead-II ECG during the echocardiogram. If an arrhythmia or electrical aberrancy was noted during a single-lead ECG, a 6-lead ECG was performed to further characterize the arrhythmia. Any tachy- or bradyarrhythmia, ventricular or atrial premature complexes, ventricular escape complexes, or electrical aberrancy (i.e., left anterior fascicular block) aside from RBBB was excluded^57^. Because RBBB can occasionally be observed in the absence of structural cardiac disease, these cats were not excluded^58^. Cats were also excluded if they had a Doppler BP $\ge$ 160 mmHg, any body cavitary effusions noted during the echocardiogram regardless of whether the effusion was cardiogenic in origin, or if they had any clinically significant abnormalities on any of the performed bloodwork. Cats were excluded if they had a significantly elevated creatinine (defined as > 1.8 mg/dL), elevated cTnI (> 90 ng/L)^59^, or elevated T4 (> 4.8 ug/dL). An elevated NTproBNP was defined as a value $\ge$ 99 pmol/L^54^. Upon completion of enrollment, cat medical records were flagged such that future visits resulting in a diagnosis of HCM would result in their removal from the study up until the time of writing.

#### Blood pressure

In cohort 1, Doppler BP measurements were performed in all cats as previously described^60^. Briefly, BP readings were performed in a quiet room. Cats were gently restrained in right lateral recumbency, and a cuff size with a width that covered approximately 30–40% of the limb circumference was chosen. The first measurement was discarded and the calculated mean of 5 readings was recorded for analysis. Owners were present, when possible, to help alleviate any stress.

#### Echocardiography

Cats were gently restrained in right and left lateral recumbency, and routine echocardiograms (Philips EPIQ 7C, Philips Healthcare, Andover, MA) were performed by a single board-certified cardiologist (JLK) using a 12–4 mHz phased-array transducer. A continuous lead-II ECG was recorded throughout the echocardiogram. Standard two-dimensional, M-mode, color Doppler, and spectral Doppler echocardiographic images were acquired. All measurements were recorded on a digital off-cart workstation (Syngo Dynamic Workplace, Siemens Medical Solutions, Inc, Malvern, Pennsylvania) by a single investigator (JLK). The final measurement recorded was the average of three consecutive cardiac cycles whenever possible. Thickness of the LVPW and IVS at end-diastole was measured in the 2-dimensional (2D) right parasternal long axis 4-chamber (RPLx4c) and 5-chamber views (RPLx5c), as well as 2-dimensional and M-mode of the right parasternal short axis view (RPSx) at the level of the papillary muscles. Two-dimensional measurements of LV wall thickness were obtained utilizing the leading-edge-to-trailing-edge method, and M-mode measurements were obtained using the leading-edge-to-leading-edge method as previously described^5^. The maximal wall thickness obtained between both LVPW and IVS measurements was recorded. A maximal LV wall thickness $\ge$ 6 mm was considered abnormal^5^. Left atrial diameter (LAD) at ventricular end-systole was measured in the RPLx4c view and RPSx at the level of the heart base. Aortic root diameter at end-systole was measured in the RPSx view at the level of the heart base and a LA: Ao ratio was calculated as previously described^61^. A LAD in RPLx > 16 mm or a LA:Ao ratio > 1.6 signified LA enlargement^62^. Left ventricular internal dimensions at end-diastole (LVIDd) and end-systole (LVIDs) were measured from the RPSx view at the level of the papillary muscles using M-mode. A percent fractional shortening (%FS) was calculated as follows: (LVIDd-LVIDs)/LVIDd*100^5^. A maximal left ventricular outflow tract velocity (LVOT_Vmax_) was measured from the left apical 5 chamber view using continuous wave (CW) Doppler^63^. A left auricular (LAA) flow velocity was measured using pulse wave (PW) Doppler from the left cranial view after optimizing for the left auricle^64^. Pulse wave Doppler of transmitral flow and PW tissue Doppler imaging (TDI) of the mitral annulus were performed to assess for indices of diastolic function^65^. However, because of the increased incidence of diastolic dysfunction with age, this was not an exclusion criterion. Intravenous administration of butorphanol was allowed if necessary to minimize stress and allow for adequate imaging^66^. No sedation was allowed prior to measuring Doppler BP.

#### Blood sample collection and analysis

Approximately 4.5 mL of blood were obtained from each subject via a cephalic, saphenous, or jugular vein phlebotomy and aliquoted as follows: 1 mL into an EDTA blood collection tube for an in-house CBC, 0.5 mL into a lithium heparin microtainer tube to perform an in-house biochemistry panel and total T4 (HM5/Vetscan2 Abaxis, Abbott Group, Union City, CA), 1 mL into a second EDTA-containing tube in preparation for DNA isolation, and 2 mL into a no-additive blood collection tube. The final 2 mL underwent centrifugation at 2000 rpm for 15 min and the resultant serum was separated into cryovials in 0.5 mL aliquots and stored at − 80 °C. Serum for NTproBNP and cTnI were then sent to IDEXX laboratories in batch for analysis (West Sacramento, CA).

#### DNA isolation and whole genome sequencing

Whole blood samples from cats successfully enrolled in the study were prepped for WGS (Fig. 1). Whole blood in EDTA blood collection tubes were centrifuged at 2000 rpm for 15 min. Genomic DNA (gDNA) was isolated from the resultant buffy coats using Gentra Puregene Blood Kit and in accordance with the manufacturer’s protocol (QIAGEN, Hilden, Germany). Once isolated, the DNA concentration and quality were assessed using spectrophotometry (NanoDrop One/One, Thermofisher, Waltham, GA, USA) and visualization on a 1% agarose gel. Unfragmented DNA samples were selected based on an adequate 260/280 ratio of ~ 1.8 and a DNA concentration of > 50 ng/uL. Samples were stored at − 20 °C until ready for shipment for WGS (Theragen Bio Co., Ltd, Gyeonggi-do, Republic of Korea). Paired-end 150 bp DNA libraries were generated using the TruSeq Nano library kit for each qualified sample. Samples were pooled and sequenced on the Illumina NovaSeq6000 platform at ~ 30 × coverage. To estimate genetic diversity, reads for each sample were processed using the WAGS pipeline^67^. Briefly, WAGS is a snakemake GATK4 best practices wrapper in which reads were mapped to the F.catus_Fca126_mat1.0^44^ reference genome with BWA-MEM^68^ and variants were called with HaplotypeCaller in GATK4^69^, and discovered variants were annotated with Ensembl’s Variant Effect Predictor (VEP)^70^.

### Cohort 2

#### Animals

Cohort 2 included a convenience sample from apparently healthy purpose-bred research cats that were humanely euthanized via intravenous pentobarbital overdose for reasons outside of the current study and in accordance with all relevant guidelines and regulations. A total of 6 cats belonged to a colony of purpose-bred mixed breed cats harboring the A31P mutation for HCM, of which three were WT) and four were heterozygous for the mutation. An additional nine unrelated cats were utilized from a university-owned nutritional research colony and all were WT for the A31P *MYBPC3* mutation.

#### Procedures

All cats had echocardiograms as described in cohort 1 performed by a board-certified cardiologist (JLK, JAS) immediately prior to humane euthanasia to confirm absence of any cardiovascular disease. Colony cats were also deemed systemically healthy based on routine bloodwork and blood pressures performed as part of their annual wellness exams. Within 30 min post-mortem, tissues were harvested from the LVPW, IVS, and LA of 11, 14, and 13 cats, respectively (Fig. 1). Once extracted, tissues were immediately flash-frozen in liquid nitrogen, and stored at − 80 °C until ready for shipment for RNA-Seq (GENEWIZ, South Plainfield, NJ). Mature mRNA was selected using the polyA method and samples underwent quality control. Samples with an RNA Integrity Number (RIN) of > 7 were selected to be converted to cDNA. Converted samples were then processed for stranded library prep. Paired-end 150 bp cDNA libraries then underwent sequencing on the Illumina HiSeq platform at a read depth of ~ 50 million reads per sample. To measure and compare expression within and among tissues, we first removed Illumina adapter sequences and low-quality reads with Trimmomatic v 0.39 (ILLUMINACLIP:2:30:10, SLIDINGWINDOW:5:20, MINLEN:100)^71^. Reads were mapped to the F.catus_Fca126_mat1.0 reference with the splicing aware STAR v2.7.11b^72^ and read counts were measured using the embedded htseq-count for each annotated gene. We conducted DEG discovery within DESeq2 v1.42.0^73^. We first removed lowly expressed genes, per DESeq recommendations, by keeping genes that have a read count > 10 among 11 samples. We then conducted a principal component analysis (PCA) based on the variance stabilized read counts. For each tissue we used all available samples to test for genes that were differentially expressed by sex (controlling for age) and age (controlling for sex). In all cases, age was a categorical variable for either above or below five years old. RNA-Seq was successfully generated for all three tissues in eight samples. We used these eight samples for paired comparisons (design =  ~ sample + tissue) to control for inter-individual variability and test for DEGs among tissues (LA v LVPW, LA v IVS, IVS v LVPW). We considered a gene differentially expressed if the absolute value of the log2fold change was greater than 1 and the p-value adjusted for multiple tests was < 0.05.

#### Gene ontology term analysis

We used ShinyGO 0.80^74^ to identify enriched gene ontology (GO) terms and KEGG^75,76^ pathways for upregulated and downregulated DEGs in IVS and LVPW tissues compared to LA tissues, as well as upregulated DEGs in LVPW tissue of male cats compared to female cats. All protein-encoding genes were selected as the default background. Criteria selected included redundancy removal, a false discovery rate (FDR) cut-off of 0.05 and a pathway size minimum of 2 and maximum of 5000.

#### Statistics for clinical and laboratory data

Statistics were performed using commercially available software (GraphPad Prism 10.2.3, San Diego, CA). Clinical, laboratory, and echocardiographic variables for cohorts 1 and 2 were tested for normality using the D’Agostino & Pearson’s test. Descriptive statistics are reported as mean (standard deviation) and median (interquartile range) for normally and non-normally distributed data, respectively.

## Electronic supplementary material

Below is the link to the electronic supplementary material.

Supplementary Material 1

## Data Availability

All data used in the production of this manuscript are available in the NCBI’s short read archive under Bioproject PRJNA1160267. The population level wgs vcf is available at: https://doi.org/10.5061/dryad.02v6wwqff.
