# Supplementary material for: Establishing a robust genetic sequencing and gene expression data library in cardiovascularly healthy cats
Source: Sci Rep. 2025 Jul 1;15:22051. doi: 10.1038/s41598-025-05704-8 (PMC12217272; doi:10.1038/s41598-025-05704-8)
Supplement: Supplementary file 1 — Supplementary Material 1 [file 41598_2025_5704_MOESM1_ESM.docx]

Supplementary Table S1: NCBI accession numbers and clinical and labwork variables for individual cats included in cohort 1.

| **Subject Number** | **Sex/Castration Status** | **Breed** | **Age (years)** | **Body Weight (kg)** | **Cardiac Auscultation** | **Heart Rate (bpm)** | **Doppler BP (mmHg)** | **T4 (ug/dL)** | **Creat (mg/dL)** | **BUN (mg/dL)** | **NT-proBNP (pmol/L)** | **cTnI (ng/L)** | **NCBI BioSample** |
| --- | --- | --- | --- | --- | --- | --- | --- | --- | --- | --- | --- | --- | --- |
| 1 | FS | DSH | 10.5 | 4.1 | Intermittent grade II/VI right parasternal systolic murmur | 185 | 149 | 2.9 | 1.5 | 28 | 86 | 38 | SAMN43718846 |
| 2 | MC | DSH | 15.8 | 4.6 | WNL | 191 | 146 | 3 | 1.3 | 28 | 99 | 70 | SAMN43718845 |
| 3 | MC | DSH | 10.7 | 6 | Grade II/VI right parasternal systolic murmur | 217 | 128 | 3.5 | 1.2 | 27 | 24 | 37 | SAMN43718842 |
| 4 | MC | DSH | 10.9 | 6.8 | WNL | 166 | 118 | 2.9 | 1.6 | 27 | 32 | 20 | SAMN43718849 |
| 5 | FS | DSH | 12.1 | 5.2 | Grade II/VI left parasternal systolic murmur | 216 | 135 | 3.7 | 1.4 | 25 | 120 | 52 | SAMN43718850 |
| 6 | MC | DSH | 10.2 | 5.9 | WNL | 156 | 126 | 3 | 1.5 | 19 | 39 | 22 | SAMN43718851 |
| 7 | MC | Siberian | 12 | 6 | WNL | 181 | 148 | 2.6 | 1.4 | 19 | 46 | 11 | SAMN43718853 |
| 8 | MC | Maine Coon mix | 13.7 | 6.5 | Grade II/VI left parasternal systolic murmur | 156 | 137 | 3.5 | 1 | 23 | 40 | 24 | SAMN43718852 |
| 9 | MC | DSH | 15.2 | 6.5 | WNL | 202 | 136 | 3.7 | 1.8 | 36 | 84 | 76 | SAMN43718847 |
| 10 | MC | Persian | 12.6 | 5.4 | WNL | 183 | 140 | 2.4 | 1.3 | 22 | 49 | 26 | SAMN43718843 |
| 11 | MC | DSH | 11 | 6.4 | WNL | 216 | 118 | 2.6 | 0.9 | 20 | 32 | 18 | SAMN43718844 |
| 12 | FS | DLH | 11.1 | 3.8 | WNL | 150 | 142 | 3 | 1.1 | 18 | 32 | 35 | SAMN43718848 |
| 13 | FS | DSH | 12 | 5.7 | WNL | 194 | 144 | 3.4 | 1.3 | 19 | 24 | 18 | SAMN43718854 |
| 14 | FS | DSH | 10.6 | 3.1 | WNL | 184 | 141 | 3 | 1.1 | 22 | 30 | 14 | SAMN43718855 |
| 15 | FS | DSH | 12.1 | 6.2 | Grade III/VI right parasternal systolic murmur | 177 | 135 | 3.4 | 1.5 | 33 | 48 | 64 | SAMN43718856 |
| 16 | FS | DSH | 12.1 | 4.6 | WNL | 154 | 138 | 4.2 | 1.7 | 21 | 63 | 33 | SAMN43718857 |
| 17 | FS | Siamese cross | 10.8 | 4.7 | WNL | 190 | 122 | 3.3 | 1.5 | 30 | 57 | 24 | SAMN43718858 |
| 18 | FS | DLH | 12.7 | 5.2 | Grade II/VI right parasternal systolic murmur | 200 | 158 | 3.2 | 1.3 | 26 | 142 | 44 | SAMN43718859 |

T4, thyroxine; BUN, blood urea nitrogen; NT-proBNP, N-terminal B-type natriuretic peptide; cTnI, cardiac troponin I; DSH, domestic shorthair; DLH, domestic longhair;WNL, within normal limits.

Supplementary Table S2: Echocardiographic variables of individuals cat included in cohort 1.

| **Subject Number** | **2D** | | | | **M-Mode** | | | **Diastolic LV Wall Thickness** | | | **Doppler** | | | | | **ECG Abnormalities** |
| --- | --- | --- | --- | --- | --- | --- | --- | --- | --- | --- | --- | --- | --- | --- | --- | --- |
|  | **RPLx4c LAD (mm)** | **RPSx LA (mm)** | **RPSx Ao (mm)** | **RPSx LA:Ao** | **LVIDd (mm)** | **LVIDs (mm)** | **%FS** | **IVSd Max (mm)** | **LVPWd Max (mm)** | **Overall Max MWT (mm)** | **LVOT Vmax (m/s)** | | **LAA Flow Vel (cm/s)** | **Spectral Doppler**  **Transmitral Flow**  **E:A ratio** | **PW TDI**  **MV Annulus**  **E':A' ratio** |  |
| 1 | 14.9 | 11.8 | 9.5 | 1.24 | 14.7 | 5.2 | 64.6 | 5.19 | 4.73 | 5.19 | 1.12 | | 88.63 | Fused E:A | Fused E':A' | RBBB |
| 2 | 11.6 | 12.6 | 9.2 | 1.37 | 15.7 | 8.6 | 45.2 | 5.79 | 4.74 | 5.79 | 0.92 | | 66.69 | Fused E:A | Fused E':A' | None |
| 3 | 15.4 | 11.5 | 9.9 | 1.16 | 13.8 | 4.3 | 68.8 | 5.72 | 5.87 | 5.87 | 1.01 | | 44.75 | Fused E:A | Fused E':A' | None |
| 4 | 15.2 | 14.2 | 9.7 | 1.46 | 15.9 | 5.1 | 67.9 | 5.85 | 5.86 | 5.86 | 0.76 | | 27.15 | Fused E:A | Fused E':A' | None |
| 5 | 13.4 | 12 | 9 | 1.33 | 13.3 | 4.2 | 68.4 | 5.95 | 5.8 | 5.95 | 1.12 | | 74.59 | Fused E:A | Fused E':A' | None |
| 6 | 13.9 | 14.3 | 10.1 | 1.42 | 15.8 | 6.5 | 58.9 | 5.72 | 5.82 | 5.82 | - | | 77.57 | 0.76 | Fused E':A' | None |
| 7 | 14.8 | 14.5 | 10.2 | 1.42 | 15.3 | 6.4 | 58.2 | 5.01 | 5.01 | 5.01 | 0.64 | | 36.67 | 1.25 | 1.2 (medial)  1.76 (lateral) | None |
| 8 | 14 | 14.1 | 10.2 | 1.38 | 16.3 | 8.6 | 47.2 | 5.43 | 5.85 | 5.85 | 1.43 | | 58.79 | Fused E:A | 0.75 (medial)  1.35 (lateral) | None |
| 9 | 13.2 | 12.4 | 10.2 | 1.22 | 16 | 6.3 | 60.6 | 5.49 | 5.59 | 5.59 | 0.99 | | 90.38 | Fused E:A | Fused E':A' | None |
| 10 | 12.3 | 12.4 | 9.9 | 1.25 | 12.5 | 4.2 | 66.4 | 5.84 | 5.65 | 5.84 | 0.73 | | 42.12 | 1.17 | Fused E':A' | None |
| 11 | 12.6 | 12.6 | 8.8 | 1.43 | 14.6 | 7.1 | 51.4 | 4.14 | 5.6 | 5.6 | 0.83 | | 35.26 | Fused E:A | Fused E':A' | None |
| 12 | 13.7 | 11.6 | 8.8 | 1.32 | 16 | 9.9 | 38.1 | 4.26 | 3.9 | 4.26 | - | | 75.9 | 1.08 | 0.82 (medial)  Fused E':A' (lateral) | None |
| 13 | 13.6 | 11.3 | 8.5 | 1.33 | 13.9 | 8.2 | 41.0 | 5.05 | 4.94 | 5.05 | 0.77 | | 35.41 | 1.23 | Fused E':A' | None |
| 14 | 12.6 | 11.7 | 8.7 | 1.34 | 13.3 | 6.4 | 51.9 | 5.84 | 4.82 | 5.84 | 0.67 | | 48.97 | 1.08 | 0.65 (medial)  0.7 (lateral) | None |
| 15 | 13.1 | 12 | 10.1 | 1.19 | 17.9 | 7.5 | 58.1 | 5.8 | 5.92 | 5.92 | 1.04 | | 59.23 | 0.94 | 0.6 (medial)  Fused E':A' (lateral) | RBBB |
| 16 | 10.6 | 11 | 9.3 | 1.18 | 15.9 | 8.5 | 46.5 | 4.84 | 4.76 | 4.84 | 0.69 | 52.21 | | 0.77 | 0.53 (medial)  1 (lateral) | None |
| 17 | 13 | 12.1 | 8.8 | 1.38 | 12.4 | 3.6 | 71.0 | 5.64 | 5.01 | 5.64 | 0.77 | 68.01 | | Fused E:A | Fused E':A' | None |
| 18 | 9.9 | 11.2 | 8.5 | 1.32 | 14.5 | 7.1 | 51.0 | 5.85 | 5.14 | 5.85 | 0.89 | 52.9 | | Fused E:A | Fused E':A' | None |

LAD, left atrial diameter; LA, left atrium; Ao, aortic root; LVIDd, left ventricular internal dimension at end-diastole; LVIDs, left ventricular internal dimension at end-systole; FS, fractional shortening; IVSd, interventricular septum at end-diastole; LVPWd, left ventricular free wall at end-diastole; MWT, myocardial wall thickness; LVOT, left ventricular outflow tract, LAA, left atrial appendage; RPLx4c, right parasternal long axis four chamber; RPSx, right parasternal short axis; MM, M-mode; V_max_, maximal velocity; PW TDI, pulse wave tissue Doppler imaging; RBBB, right bundle branch block.

Supplementary Table S3: Signalment, genotype, available tissues, and NCBI transcript accession numbers for each of the 15 purpose-bred cats in cohort 2.

| **Subject Number** | **Breed** | **Colony** | **Age at Time of Death (years)** | **M/F/Castrated/Non-Castrated** | **Body Weight (kg)** | **Genotype of A31P *MYBPC3* mutation** | **Tissues Available (flash frozen)** | **NCBI BioSample** |
| --- | --- | --- | --- | --- | --- | --- | --- | --- |
| 1 | DSH | MYBPC3 A31P Cat Colony | 7.3 | MI | 4.1 | WT | LVPW, IVS | SAMN43718867 |
| 2 | DSH | Nutrition Cat Colony | 6.9 | MI | 4.5 | WT | LVPW, IVS, LA | SAMN43718860 |
| 3 | DSH | MYBPC3 A31P Cat Colony | 9.9 | FI | 4.36 | HET | LVPW, IVS, LA | SAMN43718863 |
| 4 | DSH | MYBPC3 A31P Cat Colony | 11.2 | FS | 3.17 | HET | LVPW, LA | SAMN43718864 |
| 5 | DSH | MYBPC3 A31P Cat Colony | 1.7 | MI | 4.4 | HET | LVPW, IVS | SAMN43718868 |
| 6 | DSH | Nutrition Cat Colony | 3.4 | MI | - | - | LVPW, IVS, LA | SAMN43718869 |
| 7 | DSH | Nutrition Cat Colony | 3.7 | MI | - | - | LVPW, IVS, LA | SAMN43718870 |
| 8 | DSH | Nutrition Cat Colony | 8.4 | MI | - | - | LVPW, IVS, LA | SAMN43718871 |
| 9 | DSH | Nutrition Cat Colony | 7.2 | FI | - | - | LVPW, IVS, LA | SAMN43718872 |
| 10 | DSH | Nutrition Cat Colony | 9.1 | FI | - | - | LVPW, IVS, LA | SAMN43718873 |
| 11 | DSH | MYBPC3 A31P Cat Colony | 5.5 | MI | 5.68 | HET | LVPW, IVS, LA | SAMN43718865 |
| 12 | DSH | Nutrition Cat Colony | 3.3 | MI | 3.8 | WT | IVS, LA | SAMN43718862 |
| 13 | DSH | Nutrition Cat Colony | 3.4 | MI | 4.1 | WT | IVS, LA | SAMN43718874 |
| 14 | DSH | MYBPC3 A31P Cat Colony | 7.8 | MI | 4.78 | WT | IVS, LA | SAMN43718866 |
| 15 | DSH | Nutrition Cat Colony | 2.9 | FI | 2.9 | WT | IVS, LA | SAMN43718861 |

MYBPC3, myosin binding protein C3; DSH, domestic shorthair; HET, heterozygous; WT, wildtype; LVPW, left ventricular posterior wall, IVS, interventricular septum, LA, left atrium; MI, male intact; FS, female spayed; FI, female intact

Supplementary Table S4: Echocardiographic variables of each of the 15 purpose-bred cats in cohort 2.

| **Subject Number** | **2D** | | | | **M-Mode** | | | **Diastolic LV Wall Thickness** | | | **Doppler** | |
| --- | --- | --- | --- | --- | --- | --- | --- | --- | --- | --- | --- | --- |
|  | **RPLx4c LAD (mm)** | **RPSx LA (mm)** | **RPSx Ao (mm)** | **RPSx LA:Ao** | **LVIDd**  **(mm)** | **LVIDs (mm)** | **%FS** | **IVSd Max (mm)** | **LVPWd Max (mm)** | **Overall Max MWT (mm)** | **LVOT Vmax (m/s)** | **LAA Flow Vel (cm/s)** |
| 1 | 11.6 | 10.4 | 8.6 | 1.2 | 14.5 | 6.7 | 53.8 | 5.6 | 5 | 5.6 | 1.03 | 51.61 |
| 2 | - | - | - | - | 13.8 | 7.9 | 42.8 | 4.4 | 5.8 | 5.8 | 0.67 | - |
| 3 | 11.9 | 11.6 | 9.6 | 1.2 | 13.7 | 7.3 | 46.7 | 5.85 | 4.91 | 5.85 | 0.56 | 49.52 |
| 4 | 9.9 | 11.6 | 8.6 | 1.3 | 10.9 | 3.8 | 65.1 | 5.5 | 5.7 | 5.7 | 0.71 | 49.41 |
| 5 | 14.4 | 10.8 | 8.2 | 1.3 | 14.2 | 9.8 | 31.0 | 5.51 | 4.64 | 5.51 | 0.8 | 31.66 |
| 6 | 12.3 | 9.3 | 7.2 | 1.3 | 15 | 10.4 | 30.7 | 5.03 | 4.7 | 5.03 | 0.65 | 35.73 |
| 7 | 14.2 | 13.5 | 9.6 | 1.4 | 17 | 10 | 41.2 | 5.36 | 5.55 | 5.55 | 0.82 | 52.06 |
| 8 | 13 | 12.4 | 8.6 | 1.4 | 18.5 | 10.6 | 42.7 | 5.23 | 4.8 | 5.23 | - | 31.32 |
| 9 | 12.1 | 9.6 | 6.9 | 1.4 | 12.4 | 6.4 | 48.4 | 5.59 | 5.41 | 5.59 | 0.53 | 60 |
| 10 | 12.3 | 11.8 | 8.2 | 1.4 | 14.9 | 9.3 | 37.6 | 5.49 | 5.29 | 5.49 | 0.73 | 30.44 |
| 11 | 13.6 | 13.8 | 10.3 | 1.3 | 16.2 | 9.2 | 43.2 | 5.1 | 5.2 | 5.2 | 0.73 | 36.17 |
| 12 | 13.8 | - | - | - | 11.2 | 4.5 | 59.8 | 4.69 | 5.1 | 5.1 | - | - |
| 13 | 14 | 12.2 | 7.7 | 1.6 | - | - | - | 4.92 | 5.44 | 5.44 | 0.79 | - |
| 14 | 10.9 | 11.6 | 9.4 | 1.2 | 13.2 | 6.9 | 47.7 | 5.5 | 5.8 | 5.8 | 0.75 | 59.11 |
| 15 | 10.8 | - | - | - | 8.3 | 4 | 51.8 | 4.1 | 4.8 | 4.8 | 0.78 | - |

LAD, left atrial diameter; LA, left atrium; Ao, aortic root; LVIDd, left ventricular internal dimension at end-diastole; LVIDs, left ventricular internal dimension at end-systole; FS, fractional shortening; IVSd, interventricular septum at end-diastole; LVPWd, left ventricular free wall at end-diastole; MWT, myocardial wall thickness; LVOT, left ventricular outflow tract, LAA, left atrial appendage; RPLx4c, right parasternal long axis four chamber; RPSx, right parasternal short axis; MM, M-mode; V_max_, maximal velocity.

Supplementary Table S5: Differentially expressed genes within interventricular septal tissue of cats $\geq$5 years of age when compared to cats <5 years of age. Numbers of DEG represent the log2 of the first tissue listed over the second. A gene was considered differentially expressed when the log2fold change was greater than 1 with a p-value adjusted for multiple tests of <0.05. A log2fold change > 0 indicates a gene is upregulated and a log2fold change <0 indicates a gene is downregulated in cats $\geq$ 5 years of age with respect to cats <5 years of age.

|  | **Gene Name** | **baseMean** | **Log2FoldChange** | **Adjusted p value** | **Biotype** |
| --- | --- | --- | --- | --- | --- |
| **Upregulated** | PAK5 | 28.10 | 5.36 | 4.65E-03 | protein_coding |
| **Downregulated** | LOC123385212 | 29.12 | -2.45 | 2.67E-02 | lncRNA |

Supplementary Table S6: Differentially expressed genes within left ventricular free wall tissue of cats $\geq$5 years of age when compared to cats <5 years of age. Numbers of DEG represent the log2 of the first tissue listed over the second. A gene was considered differentially expressed when the log2fold change was greater than 1 with a p-value adjusted for multiple tests of <0.05. A log2fold change > 0 indicates a gene is upregulated and a log2fold change <0 indicates a gene is downregulated in cats $\geq$ 5 years of age with respect to cats <5 years of age.

|  | **Gene Name** | **baseMean** | **Log2FoldChange** | **Adjusted p value** | **Biotype** |
| --- | --- | --- | --- | --- | --- |
| **Upregulated** | LYVE1 | 153.73 | 2.73 | 1.10E-03 | protein_coding |
|  | RXFP1 | 141.66 | 1.82 | 1.10E-03 | protein_coding |
|  | FOLR2 | 61.72 | 2.85 | 2.21E-03 | protein_coding |
|  | LRAT | 35.55 | 4.45 | 9.76E-03 | protein_coding |
|  | CSF1R | 280.98 | 2.36 | 1.60E-02 | protein_coding |
|  | LOC102902635 | 267.56 | 1.55 | 1.73E-02 | protein_coding |
|  | LCP1 | 113.73 | 2.14 | 1.89E-02 | protein_coding |
|  | GPR34 | 40.72 | 2.71 | 1.91E-02 | protein_coding |
|  | C1QC | 86.63 | 2.37 | 2.46E-02 | protein_coding |
|  | CD163 | 283.69 | 2.45 | 2.46E-02 | protein_coding |
|  | LOC101087010 | 78.58 | 3.21 | 2.46E-02 | protein_coding |
|  | LOC101092190 | 88.96 | 2.78 | 2.46E-02 | protein_coding |
|  | C1R | 1120.54 | 1.26 | 3.95E-02 | protein_coding |
|  | CERS6 | 167.20 | 1.10 | 4.46E-02 | protein_coding |
|  | C1S | 705.74 | 1.45 | 4.94E-02 | protein_coding |
|  | PAQR9 | 83.37 | 2.03 | 4.94E-02 | protein_coding |
| **Downregulated** | LOC102899177 | 308.72 | -1.69 | 4.85E-03 | lncRNA |
|  | LOC109500514 | 82.67 | -2.18 | 1.09E-02 | lncRNA |

Supplementary Table S7: Differentially expressed genes within left atrial tissue of cats $\geq$5 years of age when compared to cats <5 years of age. Numbers of DEG represent the log2 of the first tissue listed over the second. A gene was considered differentially expressed when the log2fold change was greater than 1 with a p-value adjusted for multiple tests of <0.05. A log2fold change > 0 indicates a gene is upregulated and a log2fold change <0 indicates a gene is downregulated in cats $\geq$ 5 years of age with respect to cats <5 years of age.

|  | **Gene Name** | **baseMean** | **Log2FoldChange** | **Adjusted p value** | **Biotype** |
| --- | --- | --- | --- | --- | --- |
| **Upregulated** | SRD5A2 | 27.71 | 2.12 | 9.76E-03 | protein_coding |
|  | LOC109491424 | 11.30 | 5.42 | 3.82E-02 | lncRNA |

Supplementary Table S8: Differentially expressed genes within interventricular septal tissue of male cats when compared to female cats. Numbers of DEG represent the log2 of the first tissue listed over the second. A gene was considered differentially expressed when the log2fold change was greater than 1 with a p-value adjusted for multiple tests of <0.05. A log2fold change > 0 indicates a gene is upregulated and a log2fold change <0 indicates a gene is downregulated in male cats with respect to female cats.

|  | **Gene Name** | **baseMean** | **Log2FoldChange** | **Adjusted p value** | **Biotype** |
| --- | --- | --- | --- | --- | --- |
| **Upregulated** | LOC101083316 | 231.98 | 3.78 | 1.04E-47 | protein_coding |
|  | GPR143 | 119.10 | 2.23 | 5.25E-09 | protein_coding |
|  | KIAA1210 | 33.25 | 3.38 | 3.00E-04 | protein_coding |
|  | AURKB | 18.24 | 2.11 | 5.45E-03 | protein_coding |
|  | CD300LG | 489.30 | 1.11 | 3.94E-02 | protein_coding |
| **Downregulated** | LOC109496543 | 3636.25 | -5.76 | 4.00E-178 | lncRNA |
|  | LOC111559513 | 47.43 | -1.88 | 2.33E-02 | lncRNA |

Supplementary Table S9: Top 25 upregulated and downregulated differentially expressed genes within left ventricular free wall tissue of male cats when compared to female cats. Only a total of 9 genes were downregulated. Numbers of DEG represent the log2 of the first tissue listed over the second. A gene was considered differentially expressed when the log2fold change was greater than 1 with a p-value adjusted for multiple tests of <0.05. A log2fold change > 0 indicates a gene is upregulated and a log2fold change <0 indicates a gene is downregulated in male cats with respect to female cats.

| **Upregulated** | **Gene Name** | **baseMean** | **Log2FoldChange** | **Adjusted p value** | **Biotype** |
| --- | --- | --- | --- | --- | --- |
|  | LOC101083316 | 173.72 | 3.31 | 1.04E-04 | protein_coding |
|  | F13A1 | 167.74 | 2.79 | 2.00E-03 | protein_coding |
|  | GPR34 | 40.72 | 2.82 | 2.86E-03 | protein_coding |
|  | LYVE1 | 153.73 | 2.15 | 1.43E-02 | protein_coding |
|  | LOC102902635 | 267.56 | 1.46 | 1.47E-02 | protein_coding |
|  | OPCML | 48.28 | 1.51 | 1.47E-02 | protein_coding |
|  | PTPRC | 43.99 | 2.18 | 1.70E-02 | protein_coding |
|  | ARHGEF26 | 127.44 | 0.96 | 1.90E-02 | protein_coding |
|  | LOC123382755 | 33.60 | 3.07 | 1.90E-02 | lncRNA |
|  | MPEG1 | 68.00 | 2.59 | 1.97E-02 | protein_coding |
|  | KBTBD13 | 332.77 | 1.49 | 2.37E-02 | protein_coding |
|  | C1R | 1120.54 | 1.19 | 2.45E-02 | protein_coding |
|  | LOC101092190 | 88.96 | 2.51 | 2.45E-02 | protein_coding |
|  | LOC105259729 | 34.82 | 1.86 | 2.45E-02 | protein_coding |
|  | SNAI3 | 30.62 | 3.64 | 2.45E-02 | protein_coding |
|  | CD163 | 283.69 | 2.19 | 2.63E-02 | protein_coding |
|  | C1QC | 86.63 | 2.08 | 2.99E-02 | protein_coding |
|  | HAVCR1 | 41.30 | 2.35 | 2.99E-02 | protein_coding |
|  | KIAA1210 | 15.34 | 3.51 | 2.99E-02 | protein_coding |
|  | C3AR1 | 25.62 | 2.41 | 3.21E-02 | protein_coding |
|  | DNASE1L3 | 42.56 | 1.46 | 3.44E-02 | protein_coding |
|  | LOC123382756 | 28.38 | 3.07 | 3.85E-02 | lncRNA |
|  | SHROOM2 | 129.94 | 1.28 | 3.87E-02 | protein_coding |
|  | CD209 | 56.51 | 2.37 | 3.91E-02 | protein_coding |
|  | PDGFRA | 877.65 | 1.28 | 4.12E-02 | protein_coding |
| **Downregulated** | LOC102899177 | 308.72 | -1.71 | 1.00E-03 | lncRNA |
|  | ELL2 | 455.78 | -1.09 | 1.97E-02 | protein_coding |
|  | CSRNP3 | 48.65 | -3.13 | 2.45E-02 | protein_coding |
|  | LOC123379126 | 26.17 | -2.89 | 2.99E-02 | lncRNA |
|  | MEOX1 | 343.41 | -1.06 | 2.99E-02 | protein_coding |
|  | TIPARP | 976.26 | -1.04 | 3.18E-02 | protein_coding |
|  | PLA2G5 | 74.32 | -1.02 | 3.85E-02 | protein_coding |
|  | LOC109499490 | 951.05 | -1.12 | 4.58E-02 | lncRNA |
|  | PVR | 287.95 | -1.13 | 4.67E-02 | protein_coding |

Supplementary Table S10: Differentially expressed genes within left atrial tissue of male cats when compared to female cats. Numbers of DEG represent the log2 of the first tissue listed over the second. A gene was considered differentially expressed when the log2fold change was greater than 1 with a p-value adjusted for multiple tests of <0.05. A log2fold change > 0 indicates a gene is upregulated and a log2fold change <0 indicates a gene is downregulated in male cats with respect to female cats.

|  | **Gene Name** | **baseMean** | **Log2FoldChange** | **Adjusted p value** | **Biotype** |
| --- | --- | --- | --- | --- | --- |
| **Upregulated** | LOC101083316 | 392.58 | 3.85 | 2.82E-30 | protein_coding |
|  | GPR143 | 129.68 | 1.86 | 3.15E-02 | protein_coding |
| **Downregulated** | LOC109496543 | 6497.16 | -6.37 | 6.04E-135 | lncRNA |
|  | LOC123384967 | 27.74 | -2.39 | 1.24E-03 | lncRNA |

Supplementary Table S11: Top 25 upregulated differentially expressed genes within left atrial (LA) tissue when compared to interventricular septal (IVS) tissue of cardiovascularly normal cats. Numbers of DEG represent the log2 of the first tissue listed over the second. A gene was considered differentially expressed when the log2fold change was greater than 1 with a p-value adjusted for multiple tests of <0.05. A log2fold change > 0 indicates a gene is upregulated in LA tissue compared to IVS tissue.

| **Gene Name** | **baseMean** | **Log2FoldChange** | **Adjusted p value** | **Biotype** |
| --- | --- | --- | --- | --- |
| NPTX2 | 5785.859 | 5.706486 | 1.18E-116 | protein_coding |
| WLS | 803.8053 | 2.274239 | 9.23E-105 | protein_coding |
| NR2F1 | 291.6824 | 3.925266 | 4.56E-98 | protein_coding |
| MYL4 | 76053.91 | 5.455218 | 9.75E-97 | protein_coding |
| METTL24 | 499.2818 | 1.641258 | 1.33E-96 | protein_coding |
| MYH6 | 236298.7 | 5.820435 | 2.24E-93 | protein_coding |
| PSIP1 | 1602.742 | 1.77704 | 2.49E-92 | protein_coding |
| KCNK3 | 2229.873 | 4.72928 | 1.05E-91 | protein_coding |
| ZDHHC13 | 830.8486 | 1.695281 | 5.79E-80 | protein_coding |
| PDLIM4 | 220.1673 | 4.051012 | 6.31E-72 | protein_coding |
| RERG | 794.4578 | 3.940742 | 3.64E-64 | protein_coding |
| LOC109495115 | 162.1249 | 5.679067 | 3.99E-64 | lncRNA |
| EFEMP2 | 1106.85 | 2.616696 | 2.07E-63 | protein_coding |
| TEAD2 | 1089.984 | 1.191993 | 2.50E-63 | protein_coding |
| GDPD5 | 317.7443 | 3.522825 | 5.37E-62 | protein_coding |
| SOD3 | 1088.456 | 3.516394 | 2.46E-59 | protein_coding |
| MYL7 | 105616.6 | 5.050245 | 1.89E-58 | protein_coding |
| LOC102899982 | 100.1548 | 2.824543 | 3.93E-58 | lncRNA |
| ADAMTS12 | 649.8517 | 3.127158 | 3.93E-57 | protein_coding |
| LRRC4B | 201.2071 | 4.718598 | 4.82E-57 | protein_coding |
| RAB27B | 230.534 | 6.061728 | 4.15E-54 | protein_coding |
| NXPH3 | 230.8116 | 4.297058 | 1.75E-52 | protein_coding |
| SYT12 | 95.75755 | 3.690533 | 3.55E-52 | protein_coding |
| TXNDC5 | 1972.67 | 1.782252 | 1.38E-50 | protein_coding |
| CREB3L2 | 2716.792 | 1.296567 | 2.53E-50 | protein_coding |

Supplementary Table S12: Top 25 downregulated differentially expressed genes within left atrial (LA) tissue when compared to interventricular septal (IVS) tissue of cardiovascularly normal cats. Numbers of DEG represent the log2 of the first tissue listed over the second. A gene was considered differentially expressed when the log2fold change was greater than 1 with a p-value adjusted for multiple tests of <0.05. A log2fold change <0 indicates a gene is downregulated in LA tissue compared to IVS tissue.

| **Gene Name** | **baseMean** | **Log2FoldChange** | **Adjusted p value** | **Biotype** |
| --- | --- | --- | --- | --- |
| MYL3 | 125000.7 | -5.7585 | 0 | protein_coding |
| IRX4 | 814.6399 | -6.0053 | 6.58E-257 | protein_coding |
| SPACA7 | 2252.097 | -5.139 | 9.19E-191 | protein_coding |
| KCNJ2 | 2237.623 | -3.46405 | 3.00E-150 | protein_coding |
| ANKH | 5505.746 | -1.99001 | 1.13E-144 | protein_coding |
| LOC123382228 | 274.2844 | -3.05359 | 3.12E-143 | lncRNA |
| ABCG1 | 1030.279 | -3.50088 | 6.80E-132 | protein_coding |
| EFL1 | 2319.976 | -1.54623 | 1.61E-121 | protein_coding |
| PPFIBP1 | 18972.24 | -2.56581 | 3.88E-115 | protein_coding |
| EXTL1 | 554.9234 | -4.17917 | 2.38E-101 | protein_coding |
| SLC12A7 | 11905.48 | -2.32133 | 1.90E-99 | protein_coding |
| MFSD4A | 2730.167 | -3.41504 | 1.35E-95 | protein_coding |
| LOC102901162 | 424.0284 | -2.56491 | 4.56E-92 | lncRNA |
| MYO5B | 940.6615 | -3.38028 | 2.13E-86 | protein_coding |
| CPN2 | 2276.887 | -3.70878 | 8.95E-82 | protein_coding |
| LOC109494831 | 608.4094 | -4.816 | 1.40E-78 | lncRNA |
| LOC102902331 | 224.0263 | -3.36124 | 2.87E-77 | lncRNA |
| STRN | 1858.07 | -1.73629 | 8.62E-73 | protein_coding |
| LOC109500134 | 298.2476 | -5.73292 | 1.55E-72 | lncRNA |
| ST6GALNAC1 | 411.7629 | -4.73344 | 7.56E-71 | protein_coding |
| RNF183 | 282.693 | -4.62443 | 1.38E-68 | protein_coding |
| GPT | 3488.17 | -3.56929 | 1.73E-66 | protein_coding |
| LPCAT3 | 1763.085 | -1.37529 | 2.67E-61 | protein_coding |
| FGF13 | 1135.027 | -3.49297 | 1.31E-60 | protein_coding |
| TBC1D24 | 1652.412 | -2.12751 | 3.20E-57 | protein_coding |

Supplementary Table S13: Top 25 upregulated differentially expressed genes within left atrial (LA) tissue when compared to left ventricular posterior wall (LVPW) tissue of cardiovascularly normal cats. Numbers of DEG represent the log2 of the first tissue listed over the second. A gene was considered differentially expressed when the log2fold change was greater than 1 with a p-value adjusted for multiple tests of <0.05. A log2fold change > 0 indicates a gene is upregulated in LA tissue compared to LVPW tissue.

| **Gene Name** | **baseMean** | **Log2FoldChange** | **Adjusted p value** | **Biotype** |
| --- | --- | --- | --- | --- |
| NPTX2 | 5785.859 | 6.249265 | 7.37E-139 | protein_coding |
| MYL4 | 76053.91 | 6.238642 | 1.53E-126 | protein_coding |
| MYH6 | 236298.7 | 6.160921 | 1.17E-104 | protein_coding |
| WLS | 803.8053 | 2.263391 | 2.45E-103 | protein_coding |
| NR2F1 | 291.6824 | 4.058573 | 6.72E-102 | protein_coding |
| KCNK3 | 2229.873 | 4.917488 | 1.30E-98 | protein_coding |
| PSIP1 | 1602.742 | 1.797677 | 3.48E-94 | protein_coding |
| METTL24 | 499.2818 | 1.628165 | 3.77E-94 | protein_coding |
| PDLIM4 | 220.1673 | 4.415178 | 1.15E-80 | protein_coding |
| ZDHHC13 | 830.8486 | 1.673666 | 1.44E-77 | protein_coding |
| EFEMP2 | 1106.85 | 2.852421 | 7.18E-75 | protein_coding |
| CREB3L2 | 2716.792 | 1.540782 | 1.15E-70 | protein_coding |
| RERG | 794.4578 | 4.138805 | 3.88E-70 | protein_coding |
| LOC102899982 | 100.1548 | 3.155736 | 3.97E-66 | lncRNA |
| LOC109495115 | 162.1249 | 6.220024 | 5.18E-66 | lncRNA |
| TEAD2 | 1089.984 | 1.205119 | 2.48E-64 | protein_coding |
| MYL7 | 105616.6 | 5.129303 | 2.34E-60 | protein_coding |
| GDPD5 | 317.7443 | 3.458952 | 1.45E-59 | protein_coding |
| ADAMTS12 | 649.8517 | 3.180448 | 7.30E-59 | protein_coding |
| LRRC4B | 201.2071 | 4.823383 | 3.26E-58 | protein_coding |
| SOD3 | 1088.456 | 3.474625 | 6.04E-58 | protein_coding |
| TXNDC5 | 1972.67 | 1.904845 | 1.27E-57 | protein_coding |
| RHBDL3 | 1077.796 | 5.217203 | 5.28E-57 | protein_coding |
| ZNF483 | 291.8968 | 1.799273 | 1.78E-56 | protein_coding |
| PDIA4 | 1663.933 | 1.159243 | 5.01E-56 | protein_coding |

Supplementary Table S14: Top 25 downregulated differentially expressed genes within left atrial (LA) tissue when compared to left ventricular posterior wall (LVPW) tissue of cardiovascularly normal cats. Numbers of DEG represent the log2 of the first tissue listed over the second. A gene was considered differentially expressed when the log2fold change was greater than 1 with a p-value adjusted for multiple tests of <0.05. A log2fold change <0 indicates a gene is downregulated in LA tissue compared to LVPW tissue.

| **Gene Name** | **baseMean** | **Log2FoldChange** | **Adjusted p value** | **Biotype** |
| --- | --- | --- | --- | --- |
| MYL3 | 125000.7 | -5.89585 | 0 | protein_coding |
| IRX4 | 814.6399 | -6.13093 | 6.93E-268 | protein_coding |
| SPACA7 | 2252.097 | -5.682 | 9.05E-234 | protein_coding |
| LOC123382228 | 274.2844 | -3.23907 | 1.54E-161 | lncRNA |
| ABCG1 | 1030.279 | -3.74521 | 3.95E-151 | protein_coding |
| ANKH | 5505.746 | -2.01331 | 3.95E-148 | protein_coding |
| KCNJ2 | 2237.623 | -3.37734 | 7.10E-143 | protein_coding |
| LOC102901162 | 424.0284 | -2.90259 | 1.56E-118 | lncRNA |
| EXTL1 | 554.9234 | -4.46509 | 5.69E-116 | protein_coding |
| EFL1 | 2319.976 | -1.50513 | 5.80E-115 | protein_coding |
| SLC12A7 | 11905.48 | -2.40242 | 1.35E-106 | protein_coding |
| MFSD4A | 2730.167 | -3.5859 | 1.96E-105 | protein_coding |
| PPFIBP1 | 18972.24 | -2.39578 | 1.87E-100 | protein_coding |
| LOC109494831 | 608.4094 | -5.3287 | 1.91E-96 | lncRNA |
| LOC102902331 | 224.0263 | -3.72909 | 1.02E-95 | lncRNA |
| MYO5B | 940.6615 | -3.45372 | 3.05E-90 | protein_coding |
| CPN2 | 2276.887 | -3.81745 | 9.91E-87 | protein_coding |
| RNF183 | 282.693 | -5.12526 | 1.29E-84 | protein_coding |
| LOC102901148 | 718.1488 | -3.4017 | 3.40E-75 | lncRNA |
| GPT | 3488.17 | -3.78201 | 9.43E-75 | protein_coding |
| CDV3 | 8151.81 | -1.37607 | 3.92E-74 | protein_coding |
| ADCY10 | 454.4041 | -2.88957 | 1.41E-69 | protein_coding |
| ST6GALNAC1 | 411.7629 | -4.54905 | 2.27E-65 | protein_coding |
| LPCAT3 | 1763.085 | -1.41747 | 4.25E-65 | protein_coding |
| LOC109500134 | 298.2476 | -5.37987 | 8.82E-64 | lncRNA |

Supplementary Table S15: Up to the top 25 genes expressed overall in the left atrium (LA) tissue.

|  | Gene Symbol | Description | Mean Fragments per Million (FPM) |
| --- | --- | --- | --- |
| LA | COX1 | cytochrome c oxidase 1 | 53451.78 |
|  | COX3 | cytochrome c oxidase 3 | 28386.27 |
|  | CYTB | cytochome b | 24483.23 |
|  | COX2 | cytochrome c oxidase 2 | 23022.26 |
|  | MYH6 | myosin heavy chain 6 | 21841.41 |
|  | ND2 | NADH:ubiquinone oxidoreductase core subunit 2 | 17397.91 |
|  | ND1 | NADH:ubiquinone oxidoreductase core subunit 1 | 14776.58 |
|  | ATP6 | ATP synthase membrane subunit 6 | 13767.73 |
|  | ND4 | NADH:ubiquinone oxidoreductase core subunit 4 | 12496.12 |
|  | TNNT2 | troponin T2 | 10872.31 |
|  | MYL7 | myosin light chain 7 | 10169.57 |
|  | ND5 | NADH:ubiquinone oxidoreductase core subunit 5 | 9499.49 |
|  | MB | myogloin | 8052.95 |
|  | CKM | creatine kinase, M-type | 7863.59 |
|  | EEF1A1 | eukaryotic translation elongation factor 1 alpha 1 | 7797.01 |
|  | NPPA | natriuretic peptide a | 6481.83 |
|  | MYL4 | myosin light chain 4 | 6471.97 |
|  | ACTC1 | actin alpha cardiac muscle 1 | 5072.76 |
|  | ATP2A2 | ATPase sarcoplasmic/endoplasmic Ca2 transporting | 4806.71 |
|  | TPM1 | tropomyosin 1 | 4382.11 |
|  | TTN | titin | 4125.59 |
|  | PAM | peptidylglycine alpha-amidating monooxygenase | 3917.77 |
|  | GAPDH | Glyceraldehyde-3 phosphate dehydrogenase | 3824.85 |
|  | ND3 | NADH:ubiquinone oxidoreductase core subunit 3 | 3720.8 |
|  | DES | desmin | 3636.25 |

Supplementary Table S16: Up to the top 25 genes expressed overall in the left ventricular posterior wall (LVPW) tissue.

|  | Gene Symbol | Description | Mean Fragments per Million (FPM) |
| --- | --- | --- | --- |
| LVPW | COX1 | cytochrome c oxidase 1 | 67217 |
|  | MYH7 | myosin heavy chain 7 | 46918.8 |
|  | COX3 | cytochrome c oxidase 3 | 29498.43 |
|  | CYTB | cytochrome b | 24982.84 |
|  | COX2 | cytochrome c oxidase 2 | 22762.67 |
|  | CKM | creatine kinase, M-type | 16639.36 |
|  | ND4 | NADH:ubiquinone oxidoreductase core subunit 4 | 16074.86 |
|  | ND2 | NADH:ubiquinone oxidoreductase core subunit 2 | 15951.91 |
|  | MYL2 | myosin light chain 2 | 15391.34 |
|  | TNNT2 | troponin T2 | 15231.83 |
|  | ATP6 | ATP synthase membrane subunit 6 | 14843.04 |
|  | ND1 | NADH:ubiquinone oxidoreductase core subunit 1 | 14721.12 |
|  | ND5 | NADH:ubiquinone oxidoreductase core subunit 5 | 11095.68 |
|  | MB | myoglobin | 8546.17 |
|  | GAPDH | Glyceraldehyde-3 phosphate dehydrogenase | 7493.45 |
|  | TTN | titin | 6595.89 |
|  | TPM1 | tropomyosin 1 | 6188.87 |
|  | ACTA1 | actin alpha 1, skeletal muscle | 5580.31 |
|  | PLN | phospholamban | 4384.24 |
|  | MYL3 | myosin light chain 3 | 4281.64 |
|  | ACTC1 | actin alpha cardiac muscle 1 | 4035.1 |
|  | MYBPC3 | myosin binding protein c3 | 3981.41 |
|  | ATP2A2 | ATPase sarcoplasmic/endoplasmic Ca2 transporting | 3666.75 |
|  | DES | desmin | 3640.86 |
|  | ND3 | NADH:ubiquinone oxidoreductase core subunit 3 | 3393.29 |

Supplementary Table S17: Top 25 genes expressed overall in the interventricular septum (IVS) tissue.

|  | Gene Symbol | Description | Mean Fragments per Million (FPM) |
| --- | --- | --- | --- |
| IVS | COX1 | cytochrome c oxidase 1 | 73655.3 |
|  | MYH7 | myosin heavy chain 7 | 45377.78 |
|  | COX3 | cytochrome c oxidase 3 | 30451.59 |
|  | CYTB | cytochrome b | 25397.92 |
|  | COX2 | cytochrome c oxidase 2 | 23720.32 |
|  | ND2 | NADH:ubiquinone oxidoreductase core subunit 2 | 17795.72 |
|  | ND1 | NADH:ubiquinone oxidoreductase core subunit 1 | 16666.62 |
|  | ND4 | NADH:ubiquinone oxidoreductase core subunit 4 | 16516.01 |
|  | CKM | creatine kinase, M-type | 15957.28 |
|  | ATP6 | ATP synthase membrane subunit 6 | 14291.73 |
|  | MYL2 | myosin light chain 2 | 13543.74 |
|  | ND5 | NADH:ubiquinone oxidoreductase core subunit 5 | 13367.57 |
|  | TNNT2 | troponin T2 | 13165.05 |
|  | MB | myoglobin | 9061 |
|  | GAPDH | Glyceraldehyde-3 phosphate dehydrogenase | 7412.69 |
|  | TTN | titin | 6204.64 |
|  | TPM1 | tropomyosin 1 | 5208.89 |
|  | ACTA1 | actin alpha 1, skeletal muscle | 4472.65 |
|  | PLN | phospholamban | 4449.07 |
|  | MYBPC3 | myosin binding protein c3 | 4142.03 |
|  | MYL3 | myosin light chain 3 | 3938.7 |
|  | ATP2A2 | ATPase sarcoplasmic/endoplasmic Ca2 transporting | 3790.11 |
|  | ACTC1 | actin alpha cardiac muscle 1 | 3756.23 |
|  | DES | desmin | 3504.92 |
|  | ND3 | NADH:ubiquinone oxidoreductase core subunit 3 | 3416.96 |

Supplementary Table S18: Compilation of the top 25 genes expressed overall listed in supplementary tables 15-17. Genes are listed in alphabetical order and the locations in which they are expressed to show tissues in which there is overlap of gene expression. LA, left atrium; LVPW, left ventricular posterior wall; IVS, interventricular septum.

| Gene Symbol | Description | LA | LVPW | IVS |
| --- | --- | --- | --- | --- |
| ACTA1 | actin alpha 1, skeletal muscle |  | ✓ | ✓ |
| ACTC1 | actin alpha cardiac muscle 1 | ✓ | ✓ | ✓ |
| ATP2A2 | ATPase sarcoplasmic/endoplasmic Ca2 transporting | ✓ | ✓ | ✓ |
| ATP6 | ATP synthase membrane subunit 6 | ✓ | ✓ | ✓ |
| CKM | creatine kinase, M-type | ✓ | ✓ | ✓ |
| COX1 | cytochrome c oxidase 1 | ✓ | ✓ | ✓ |
| COX2 | cytochrome c oxidase 2 | ✓ | ✓ | ✓ |
| COX3 | cytochrome c oxidase 3 | ✓ | ✓ | ✓ |
| CYTB | cytochrome b | ✓ | ✓ | ✓ |
| DES | desmin | ✓ | ✓ | ✓ |
| EEF1A1 | eukaryotic translation elongation factor 1 alpha 1 | ✓ |  |  |
| GAPDH | Glyceraldehyde-3 phosphate dehydrogenase | ✓ | ✓ | ✓ |
| MB | myoglobin | ✓ | ✓ | ✓ |
| MYBPC3 | myosin binding protein c3 |  | ✓ | ✓ |
| MYH6 | myosin heavy chain 6 | ✓ |  |  |
| MYH7 | myosin heavy chain 7 |  | ✓ | ✓ |
| MYL2 | myosin light chain 2 |  | ✓ | ✓ |
| MYL3 | myosin light chain 3 |  | ✓ | ✓ |
| MYL4 | myosin light chain 4 | ✓ |  |  |
| MYL7 | myosin light chain 7 | ✓ |  |  |
| ND1 | NADH:ubiquinone oxidoreductase core subunit 1 | ✓ | ✓ | ✓ |
| ND2 | NADH:ubiquinone oxidoreductase core subunit 2 | ✓ | ✓ | ✓ |
| ND3 | NADH:ubiquinone oxidoreductase core subunit 3 | ✓ | ✓ | ✓ |
| ND4 | NADH:ubiquinone oxidoreductase core subunit 4 | ✓ | ✓ | ✓ |
| ND5 | NADH:ubiquinone oxidoreductase core subunit 5 | ✓ | ✓ | ✓ |
| NPPA | natriuretic peptide a | ✓ |  |  |
| PAM | peptidylglycine alpha-amidating monooxygenase | ✓ |  |  |
| PLN | phospholamban |  | ✓ | ✓ |
| TNNT2 | troponin T2 | ✓ | ✓ | ✓ |
| TPM1 | tropomyosin 1 | ✓ | ✓ | ✓ |
| TTN | titin | ✓ | ✓ | ✓ |

Supplementary Figure S1: Enriched terms for upregulated and downregulated DEG in interventricular septal tissue (IVS) when compared to left atrial (LA) tissue. The following enrichment plots are shown: A) GO KEGG pathway for upregulated DEG within the IVS, B) GO biological process (BP) for upregulated DEG within the IVS, C) GO cellular component (CC) for upregulated DEG within the IVS, C), D) GO KEGG pathway for downregulated DEG within the IVS, E) GO BP for downregulated DEG within the IVS, and E) GO CC for downregulated DEG within the IVS. The enriched terms are listed in order of statistical significance along the y-axis with those of greatest statistical significance listed at the top. Fold enrichment is listed along the x-axis (-log10[FDR]). The size of each data point is dependent on the number of total genes associated with each enriched term compared to a default background. Red and blue data points signify high or low fold enrichment, respectively.


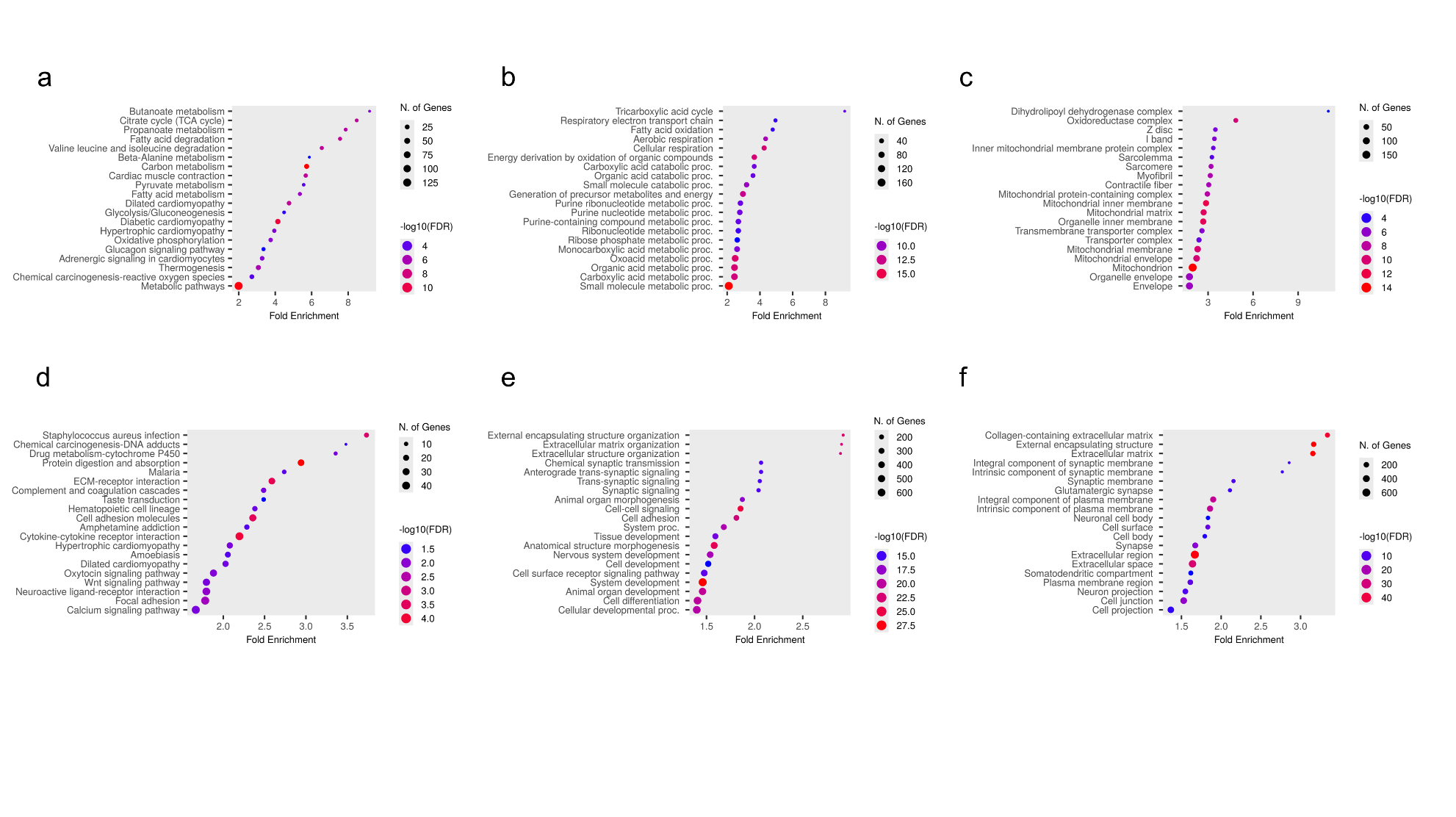


Supplementary Figure S2: Enriched terms for upregulated and downregulated DEG in the left ventricular posterior wall (LVPW) when compared to left atrial (LA) tissue. The following enrichment plots are shown: A) GO KEGG pathway for upregulated DEG within the LVPW, B) GO biological process (BP) for upregulated DEG within the LVPW, C) GO cellular component (CC) for upregulated DEG within the LVPW, D) GO KEGG pathway for downregulated DEG within the LVPW, E) GO BP for downregulated DEG within the LVPW, and F) GO CC for downregulated DEG within the LVPW. The enriched terms are listed in order of statistical significance along the y-axis with those of greatest statistical significance listed at the top. Fold enrichment is listed along the x-axis (-log10[FDR]). The size of each data point is dependent on the number of total genes associated with each enriched term compared to a default background. Red and blue data points signify high or low fold enrichment, respectively.


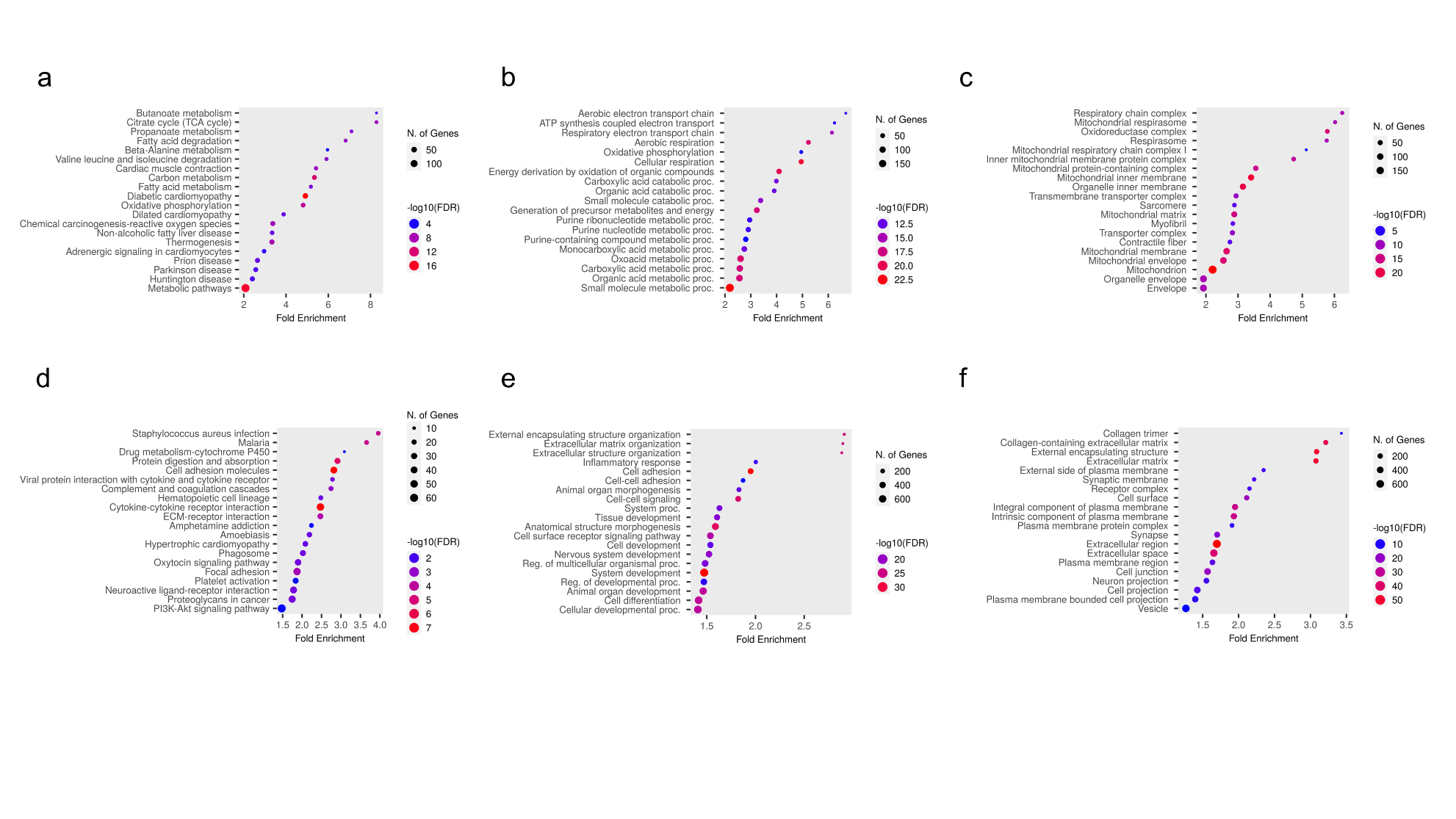


Supplementary Figure S3: Enriched terms for upregulated DEG in the left ventricular posterior wall (LVPW) in male cats when compared to female cats. The following enrichment plots are shown: A) GO KEGG pathway for upregulated DEG within the LVPW, B) GO biological process (BP) for upregulated DEG within the LVPW, and C) GO cellular component (CC) for upregulated DEG within the LVPW of male cats. The enriched terms are listed in order of statistical significance along the y-axis with those of greatest statistical significance listed at the top. Fold enrichment is listed along the x-axis (-log10[FDR]). The size of each data point is dependent on the number of total genes associated with each enriched term compared to a default background. Red and blue data points signify high or low fold enrichment, respectively.
